# IL10RB-AS1 promotes pro-caspase-1-dependent pyroptosis via regulating the oligomerization and nucleation of CARD8 C-terminal domain to suppress acute myeloid leukemia

**DOI:** 10.1038/s41419-026-09129-7

**Published:** 2026-07-31

**Authors:** Qi Pan, Bo He, Li-Bin Huang, Qian-Qian Yang, Cheng-Lin Zhou, Yan An, Xin-Yi Feng, Jian-Ping Lian, Shun-Xin Zhu, Ke-Jia Pu, Heng-Jing Huang, Chen Fang, Yi-Xuan Ma, Chang-Hao Liang, Zi-Yuan Wang, Dan Wang, Wei Huang, Yun-Meng Sun, Yue-Qin Chen, Hua Zhang, Wen-Tao Wang

**Affiliations:** 1 MOE Key Laboratory of Gene Function and Regulation, Guangdong Province Key Laboratory of Pharmaceutical Functional Genes, State Key Laboratory of Biocontrol, School of Life Sciences, Sun Yat-sen University, Guangzhou, China; 2 Institute of Laboratory Medicine, Guangdong Provincial Key Laboratory of Medical Immunology and Molecular Diagnostics, Key Laboratory of Big Data Mining and Precision Drug Design, School of Medical Technology, Guangdong Medical University, Dongguan, China; 3 Department of Hepatobiliary, and Department of Anesthesiology, Sun Yat-sen Memorial Hospital, Sun Yat-sen University, Guangzhou, China; 4 Department of Pediatrics, The First Affiliated Hospital, Sun Yat-sen University, Guangzhou, China; 5 Sun Yat-sen University Cancer Center, State Key Laboratory of Oncology in South China, Guangzhou, China

**Keywords:** Long non-coding RNAs, Cell death

## Abstract

CARD8 (caspase activation and recruitment domain-containing 8) senses intracellular danger signals, leading to inflammasome formation and pyroptosis. Recently studies have shown that CARD8 was highly expressed in hematopoietic cells, however, the mechanism by which the released C- terminal domain of CARD8 (CARD8-CT) oligomerizes and nucleates, and its role in acute myeloid leukemia (AML) treatment has not been fully understood. Here, we identified a long noncoding RNA IL10RB-AS1 as a novel regulator that can enhance the CARD8-CT oligomer assembly and promote pro-caspase-1-dependent pyroptosis in AML cells. Mechanically, IL10RB-AS1 recruits DEAD-box helicase 3, X-linked (DDX3X) to CARD8 and enhances the DDX3X-CARD8 interaction to promote CARD8-CT nucleation. This process activates the CARD8 inflammasome and triggers an ASC-independent pyroptosis response to perturbations in cellular homeostasis. We found that the C-terminal domain of DDX3X is essential for its interaction with both IL10RB-AS1 and CARD8-CT, facilitating inflammasome activation during AML cell pyroptosis. Importantly, IL10RB-AS1 is highly expressed across AML and correlates with poor outcomes. This elevated expression of IL10RB-AS1 promotes DPP8/9 inhibitor induced pro-caspase-1-dependent pyroptosis in vivo, suggesting that high IL10RB-AS1 levels may predict cellular sensitivity to these inhibitors. Our findings not only reveal a novel mechanism by which a lncRNA orchestrates CARD8-CT nucleation but also highlight new therapeutic opportunities for DPP8/9 inhibitors in hematopoietic malignancies with high IL10RB-AS1 expression.

## Background

Acute myeloid leukemia (AML) derives from malignant transformation of haematopoietic stem and myeloid progenitor cells, is the most common leukemia in adults, with a very poor prognosis [1–3]. The genetic complexity and the constant evolution of mutational landscape in AML subclones during progression contribute to the treatment resistance and high rates of relapse [1, 4, 5]. Thus, developing novel therapeutic strategies is urgently needed to improve the clinical outcomes of AML.

In the past decade, extensive studies have revealed a novel manner of cell death, pyroptosis that was induced by chemotherapeutic drugs and boost the effect of antitumor therapy [6–10]. Pattern recognition receptors (PRRs) detect danger-associated signals, assembling into inflammasomes, and trigger pyroptosis [7, 8, 10–13]. Exogenous activation of inflammasome-triggered pyroptosis induces robust antitumor immunity and produces pores in the cell membrane to kill the cancer cells [6, 12, 13]. Previous studies have shown that CARD8 (caspase activation and recruitment domain-containing 8) is a PRR that is specifically and highly expressed in hematopoietic cells, where it nucleates the inflammasome to mediate pyroptosis under certain perturbations in cellular homeostasis [14–17]. It was also reported that the CARD8 inflammasome mediates pro-caspase-1-dependent pyroptosis in AML cells upon treatment with dipeptidyl peptidase 8 and 9 (DPP8/9) inhibitors [18]. These recent findings may suggest that CARD8 inflammasome-triggered pyroptosis could be a promising strategy for AML treatment. Thus, understanding of the mechanisms underlying the formation of CARD8 inflammasomes is necessary to develop a rational basis for the design of novel therapies to treat AML through pyroptosis.

Among the diverse pyroptosis process, the nucleation of inflammasomes is a pivotal event in the most of pyroptosis process [11–14, 19, 20]. Canonical inflammasomes are composed of an activated PRR, apoptosis-associated speck-like protein containing a CARD (ASC), and caspase-1, and initiate autoproteolytic processing of caspase-1, thereby promoting the formation of gasdermin D (GSDMD) pores, cell rupture, and secretion of bioactive interleukin-1β (IL-1β) and interleukin-18(IL-18) [8, 11, 12]. Unlike canonical PRRs, CARD8 contains the function-to-find domain (FIIND)-CARD motif and nucleates inflammasome in an ASC-independent pattern [11, 14, 17, 20]. In this process, pro-caspase-1 was activated without autoproteolysis, which only cleaves GSDMD but not the inflammatory cytokines, thereby triggering a less inflammatory form of lytic cell death [11, 14, 17, 20, 21]. DPP8/9 could associate with the FIIND of CARD8 and represses inflammasome formation [14, 22, 23]. Several small-molecule inhibitors of DPP8/9, such as Val-boroPro (VbP) and CQ31, may promote CARD8’s N terminus (CARD8-N) degradation and thereby freeing its C-terminal fragment (CARD8-CT) to activate CASP1-GSDMD pathway [16, 23–25]. Although these studies have expanded our understanding of CARD8-mediated pyroptosis, the mechanism underlying the oligomerization and nucleation of the released CARD8-CT and its role on AML treatment thorough pyroptosis remains to be elucidated.

Several recent studies highlight the emerging role of long noncoding RNAs (lncRNAs) in regulating inflammasome-mediated pyroptosis across multiple diseases [26–30]. For instance, lncRNA Lfar1 knockdown significantly suppressed LPS- and IFN-γ-induced proinflammatory activation of macrophages, and inhibited LPS / Nigericin-induced NLRP3 inflammasome-mediated pyroptosis [27]. Knockdown of the X inactivation-specific transcript (XIST) could aggrandize cisplatin-induced pyroptosis in lung cancer cells by increasing the expression levels of the nucleotide-binding leucine-rich repeat pyrin domain-containing protein 3 (NLRP3), caspase-1, IL-18, and N-terminal of GSDMD [29]. Many lncRNAs dysregulated across drug-induced pyroptosis and associated with poor prognosis [27–29, 31]. We previously revealed a set of lncRNAs that regulate leukemia cell stemness and differentiation blockage [32–36]. We thus asked whether lncRNAs are also essential as regulators that orchestrates the nucleation of inflammasomes, such as CARD8-CT oligomerization and nucleation? Does this regulation benefit AML treatment through CARD8 inflammasome-meditated pyroptosis? Here, we discovered a highly expressed lncRNA IL10RB-AS1 promotes DPP8/9 inhibitors-induced pyroptosis in most AML cell lines, and inhibits AML progression in vivo. Mechanically, we firstly uncovered DEAD-box helicase 3, X-linked (DDX3X) protein interacts with CARD8-CT with its C-terminal domain, and plays a crucial role in CARD8 inflammasome-mediated pyroptosis. IL10RB-AS1 enhances the DDX3X-CARD8 interaction to promote CARD8-CT oligomer assembly and nucleation activating. This study not only reports a previously unknown mechanism of lncRNAs orchestrating the nucleation of CARD8 inflammasome, but also provides new opportunities for activating CARD8-mediated pyroptosis in hematopoietic malignancies with highly expressed IL10RB-AS1.

## Results

### IL10RB-AS1 is highly expressed in AML and associated with pyroptosis

**Fig. 1:**
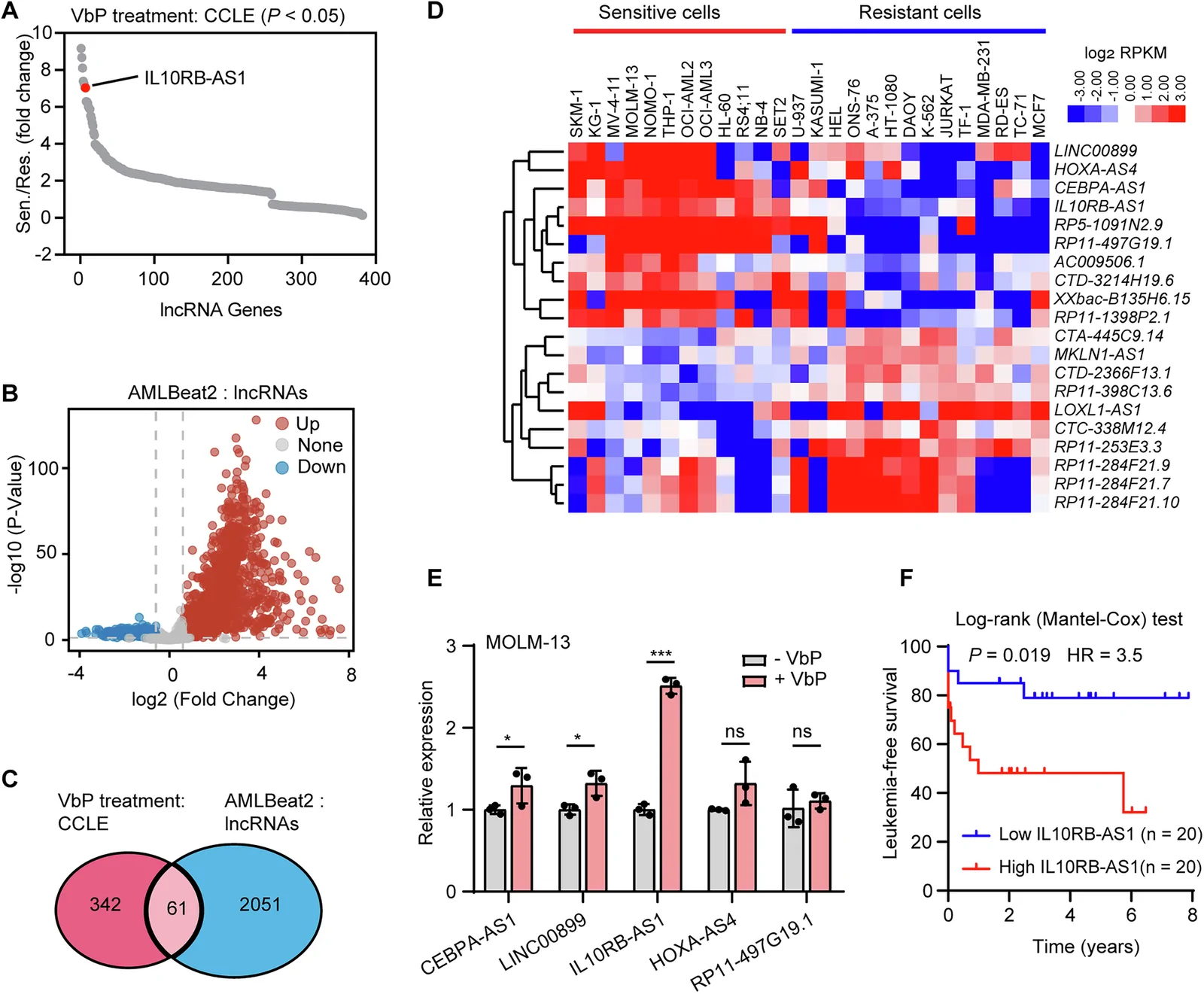
High IL10RB-AS1 expression confers VbP sensitivity and predicts poor outcomes in AML. **A** lncRNAs whose level of expression (from CCLE microarray data 2019) distinguishes between 26 cell lines that are sensitive (*n* = 12) and resistant (*n* = 14) to Val-boroPro (VbP) that identifed by Darren C. Johnson et al.^18^. *y*-axis inditates the lncRNA whose expression fold change between sensitivity and resistance cells (mean of base read > 0.5 and *P* < 0.05). The *x*-axis displays the ranking of lncRNAs based on fold change. IL10RB-AS1, whose expression level is most highly correlated with sensitivity and resistance, is shown. **B** Volcano plots of lncRNA expression of RNA-seq from AMLBeat 2.0 for the difference in AML and health control groups determined by two-tailed Student’s t-test from a linear model fit (*x*-axis). The *y* axis indicates the *P* values. The numbers of significantly downregulated (fold change [FC] < −1.5, *P* < 0.05) or upregulated (FC > 1.5, *P* < 0.05) genes were shown. Vertical dashed lines indicate cut-off of FC (1.5 or −1.5); horizontal dashed lines indicate cut-off of *P* value (0.05). **C** Venn diagram showed the dysregualted lncRNAs assciated with the sensitive to VbP in AML. **D** The 20 lncRNAs whose expression levels (from CCLE microarray data 2019) are most highly correlated with sensitivity and resistance to VbP are shown. The top ten lncRNAs are highly expressed in sensitive cells lines, and the bottom ten lncRNAs are highly expressed in resistant cell lines. **E** qRT-PCR for the top 5 lncRNAs whose expression levels are most highly correlated with sensitivity to VbP shown in **D** MOLM-13 cells with or without VbP treatment. Values are mean ± SD (*n* = 3), and unpaired two-tailed Student’s *t*-test. ns, not significant; * *P* < 0.05; *** *P* < 0.001. **F** The leukemia-free survival of patients with a high expression level of IL10RB-AS1 is less than that of patients with a low IL10RB-AS1 level in AML. *P* value and hazard ratio were calculated using the log-rank test (*n* = 40, *P* = 0.019).

Inhibitors of proteases DPP8/9, such as VbP, can activate a human CARD8 inflammasome [14, 16, 18, 22, 23]. To investigate whether lncRNAs contribute to CARD8 inflammasome-mediated pyroptosis, we firstly investigated and combined analysis of the RNA-seq data from the Cancer Cell Line Encyclopedia (CCLE) database and AMLBeat2 data set [37, 38], and identified a set of dysregulated lncRNAs (*n* = 61) that may play a pivotal role in VbP sensitivity in AML cells (Fig. 1A–C). Figure 1D listed the lncRNAs as a top 20 predictor of VbP sensitivity across the hematopoietic cell lines, and which highly expressed in various subtypes of AML cells. In addition, VbP activated CARD8 inflammasome-mediated pyroptosis in AML cells [18]; the dying cells showed swelling with characteristic large bubbles from the plasma membrane (Fig. S1A). Among the top five lncRNAs (those with expression levels most highly correlated with VbP sensitivity), IL10RB-AS1 was found remarkably induced with VbP treatment in AML cells undergoing pyroptosis (Fig. 1E), suggesting that IL10RB-AS1 may be a pivotal regulator of VbP sensitivity across AML cells, and thus was chosen for further investigation.

*IL10RB-AS1* located at Chromosome 21 and includes two exons, and transcribed a lncRNA with 466 nt in length (Fig. S1B–D). We also verified that the leukemia-free survival of AML patients with higher IL10RB-AS1 levels was less than that of patients with low IL10RB-AS1 levels (Fig. 1F), suggesting that the abundance of IL10RB-AS1 could be a potential risk factor for AML. (Detailed clinical patient information is provided in Table S1). These data together indicate that IL10RB-AS1 may could serve as a critical risk factor, and potential as a predictor for AML cells that can be suffered from DPP8/9 inhibitor-induced pyroptosis.

### IL10RB-AS1 regulates non-canonical pyroptosis in AML cells

**Fig. 2:**
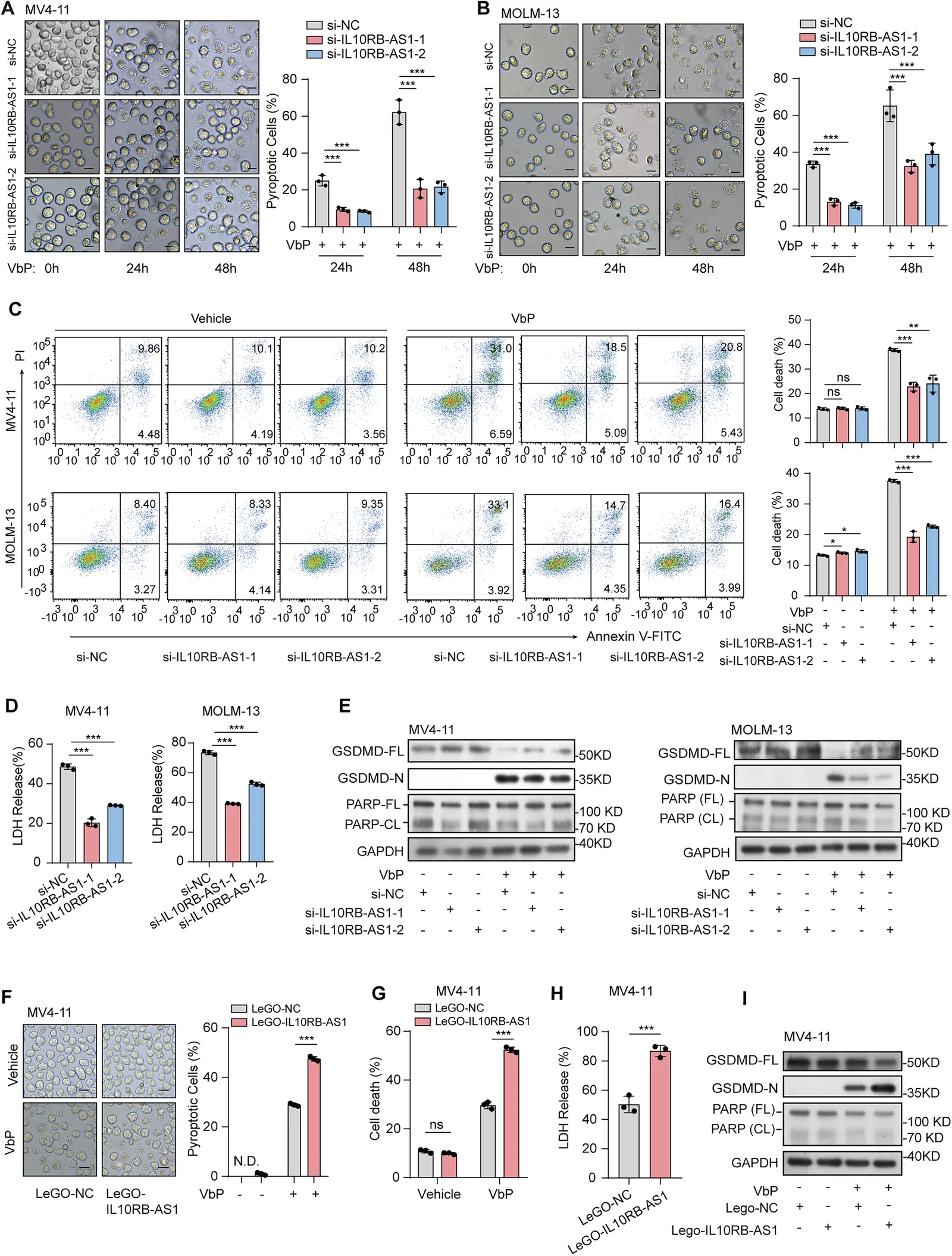
IL10RB-AS1 confers VBP-induced pyroptosis in AML cells. Knockdown of lncRNA IL10RB-AS1 attenuates cell pyroptosis in both MV4-11 (**A**) and MOLM-13 (**B**) cell lines. Representative bright-field images (left) and quantitative analysis(right) demonstrate a significant reduction in the number of pyroptotic cells (characterized by cell swelling and membrane rupture) after knockdown of IL10RB-AS1 with small interfering RNA followed by 5 µM VbP treatment for 24 and 48 hours (h). Scale bar:20 μm. **C** Flow cytometric analysis of cell death (PI+ / Annexin V+ cells) in AML cells following electrotransfection with si-NC or si-IL10RB-AS1/2 and subsequent treatment with or without 5 µM VbP. **D** LDH release from AML cells after electrotransfection with si-NC or si-IL10RB-AS1-1/2 and treatment with 5 µM VbP. **E** Western blot for the GSDMD and PARP in AML cells following electrotransfection with si-NC or si-IL10RB-AS1-1/2 and subsequent treatment with or without 5 µM VbP. GAPDH serves as loading control. FL: full-length, N: N terminus, CL cleaved. **F** Representative pyroptotic morphology (left) and quantitative analysis(right) for MV4-11 after transfected with LeGO-NC or LeGO-IL10RB-AS1 and treatment with 5 µM VbP. N.D.: no canonical pyroptotic cells in the detection. **G** Flow cytometry quantification of cell death in MV4-11 transfected with LeGO-NC or LeGO-IL10RB-AS1 and treatment with 5 µM VbP. **H** LDH release from MV4-11 transfected with LeGO-NC or LeGO-IL10RB-AS1 and treatment with 5 µM VbP. **I** Western blot for the GSDMD and PARP in MV4-11 following transfection with LeGO-NC or LeGO-IL10RB-AS1 and treatment with 5 µM VbP. GAPDH serves as loading control. FL: full-length, N: N-terminal, CL: cleaved. Values are mean ± SD (*n* = 3), one-way ANOVA in **A**–**D**, and unpaired two-tailed Student’s *t*-test in **F-H**. ns, not significant; *P < 0.05; **P < 0.01; ***P < 0.001.

To explore whether IL10RB-AS1 is required for cytotoxicity and CARD8 inflammasome-mediated pyroptosis, loss-of and gain-of-function experiments were performed. The efficiencies of knocking down IL10RB-AS1 in leukemic cells were shown in Fig. S2. Silencing IL10RB-AS1 significantly attenuated the DPP8/9 inhibitor-induced pyroptosis on all tested cell lines originated from various subtypes of AML, including decreased number of pyroptosis cells, inhibiting cell death, and reducing the release of lactate dehydrogenase (LDH) (Figs. 2A–D and S3A–C). These data indicated that IL10RB-AS1 is required for DPP8/9 inhibitor-mediated cytotoxicity in AML cells.

DPP8/9 inhibitors induce the CARD8 inflammasome-nucleating in an ASC-independent pattern [14, 16, 18]. This process activates pro-caspase-1 [14, 18, 21]. Specifically, this active pro-caspase-1 species does not mediate autoprocessing or the cleavage of inflammatory cytokines but efficiently cleave GSDMD to induce non-canonical pyroptosis [14, 15, 18, 21]. We also found that DPP8/9 inhibitor treatment can not affect the cleavage of pro-caspase-1 and the apoptotic substrate polyADP-ribose polymerase (PARP) in AML cells (Fig. S4A). Moreover, we added the caspase-1 inhibitor Belnacasan (VX‑765) to VbP-treated AML cells. VbP treatment significantly cleaved GSDMD, leading to GSDMD‑N fragment accumulation and pyroptotic cell formation; all of which were rescued by VX‑765, indicating that DPP8/9 inhibitor-activated pro-caspase-1 cleaves GSDMD and promotes pyroptosis in AML cells (Fig. S4B, C).

Knockdown of IL10RB-AS1 did not alter GSDMD mRNA levels but significantly suppressed the cleavage of the GSDMD protein upon VbP treatment (Figs. 2E and S4D, E). In contrast, IL10RB-AS1 silencing had minimal impact on the cleavage of pro-caspase-1 and PARP upon VbP treatment, indicating that it plays a limited role in apoptosis but is a key regulator of active pro-caspase-1-mediated non-canonical pyroptosis (Figs. 2E and S4F). Moreover, overexpressing of IL10RB-AS1 significantly accelerated the VbP-induced pyroptosis on AML cells, including increased number of pyroptosis cells, promoting cell death, inducing the release of LDH, and improving the cleavage of GSDMD (Figs. 2F–I and S4G, H). These data together indicated that IL10RB-AS1 expression is important for DPP8/9 inhibitor-induced pyroptosis in AML cells and even promotes the cells sensitive to VbP-induced cytotoxicity.

### IL10RB-AS1 modulates the oligomerization of CARD8-CT in CARD8-mediated pyroptosis

**Fig. 3:**
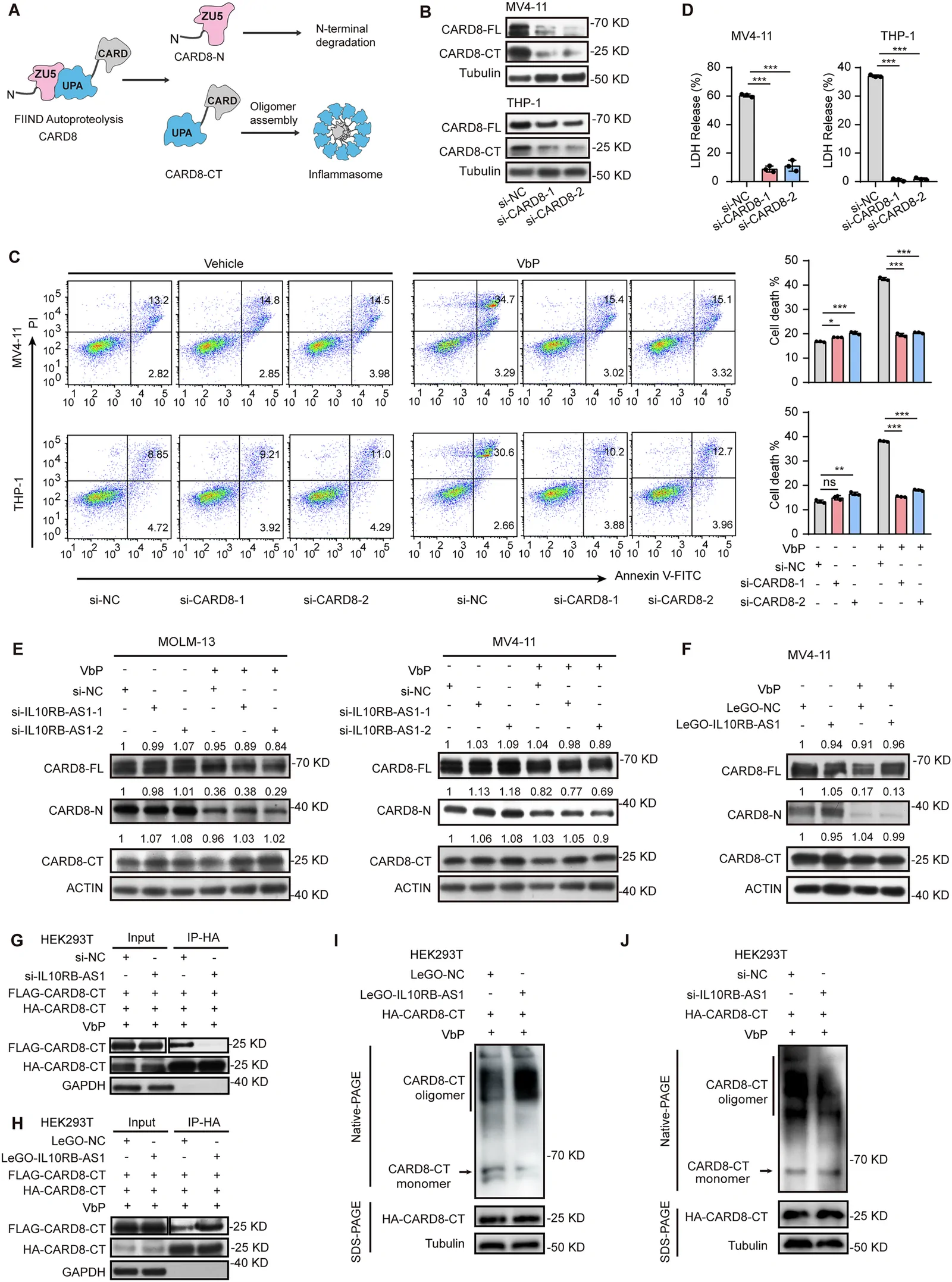
LncRNA IL10RB-AS1 regulates the nucleation of CARD8 inflammation and pyroptosis. **A** Schematic diagram illustrating the process of formation of CARD8 inflammasome. **B** Western blot showing reduced CARD8 protein levels in MV4-11 and THP-1 cells 48 hours post-knockdown by siRNA. Tubulin serves as loading control. **C** Flow cytometric analysis of cell death (PI+ / Annexin V+ cells) in AML cells following electrotransfection with si-NC or si-CARD8-1/2 and subsequent treatment with or without 5 µM VbP. **D** LDH release from AML cells after electrotransfection with si-NC or si-CARD8-1/2 and treatment with 5 µM VbP. Western blot for the CARD8 in AML cells following transfection with si-IL10RB-AS1-1/2 (**E**) or LeGO-IL10RB-AS1 (**F**) and subsequent treatment with or without 5 µM VbP. ACTIN serves as loading control. FL: full-length, N: N-terminal, CT: C-terminal. The CARD8-FL, CARD8-N, or CARD8-CT / ACTIN densitometric ratio were recorded by ImageJ. Immunoprecipitation of CARD8-C-terminal interactions in IL10RB-AS1-silencing (**G**) or -overexpressing (**H**) cells and treatment with 5 µM VbP. CARD8-CT oligomerization analyzed by native gel under IL10RB-AS1 overexpression (**I**) or knockdown (**J**). Black arrows, monomeric species. FL: full-length, N: N-terminal, CT: C-terminal domain. Values are mean ± SD (*n* = 3), one-way ANOVA in **C**, **D**. ns, not significant; *P < 0.05; **P < 0.01; ***P < 0.001.

Next, we investigated how IL10RB-AS1 regulates CARD8-mediated pyroptosis induced by DPP8/9 inhibitors in AML cells. Since the cleavage of CARD8 and inflammasome nucleation is prime to the pyroptosis [23, 39], we tested whether IL10RB-AS1 influences these initial steps (Fig. 3A). Loss-of-function of IL10RB-AS1 verified that CARD8 is required for DPP8/9 inhibitor-induced pyroptosis in AML cells (Fig. 3B–D). CARD8 autoprocesses into noncovalently associated CARD8-N and CARD8-CT fragments and binds the cellular dipeptidyl peptidases DPP8 and 9 (DPP8/9). The DPP8/9 inhibitor VbP can induce the proteasome-mediated CARD8-N degradation and thereby liberate the inflammasome-CARD8-CT [16, 23, 40, 41]. Our observation has also shown that VbP significantly promoted the CARD8-N degradation in AML cells; however, manipulating IL10RB-AS1 expression has a limited effect on this process (Fig. 3E, F). Moreover, manipulating IL10RB-AS1 expression slightly changed the levels of CARD8-CT and full length of CARD8 (Fig. 3E, F).

As oligomerization of the CARD8-CT domain is essential for inflammasome activation [23, 39], we next asked if IL10RB-AS1 promotes pyroptosis by nucleating the inflammasome via this mechanism (Fig. 3A). The result showed that IL10RB-AS1 knockdown severely impaired the interaction between Flag- and HA-tagged CARD8-CT, while its overexpression enhanced this interaction, suggesting a pivotal role of IL10RB-AS1 in CARD8-CT oligomer assembly (Fig. 3G, H). Notably, in a Native-PAGE assay, IL10RB-AS1 overexpression significantly promoted the CARD8-CT oligomerization in VbP-treated cells, as evidenced by the accumulation of high-molecular-weight species (Fig. 3I). Conversely, its knockdown suppressed the formation of these higher-order oligomers (Fig. 3J). Thus, IL10RB-AS1 may be critical for the CARD8-CT oligomer assembly, nucleating and priming the CARD8 inflammasome to induce pyroptosis in AML cells.

### IL10RB-AS1 activates CARD8 inflammasome via interacting with DDX3X in AML cells

**Fig. 4:**
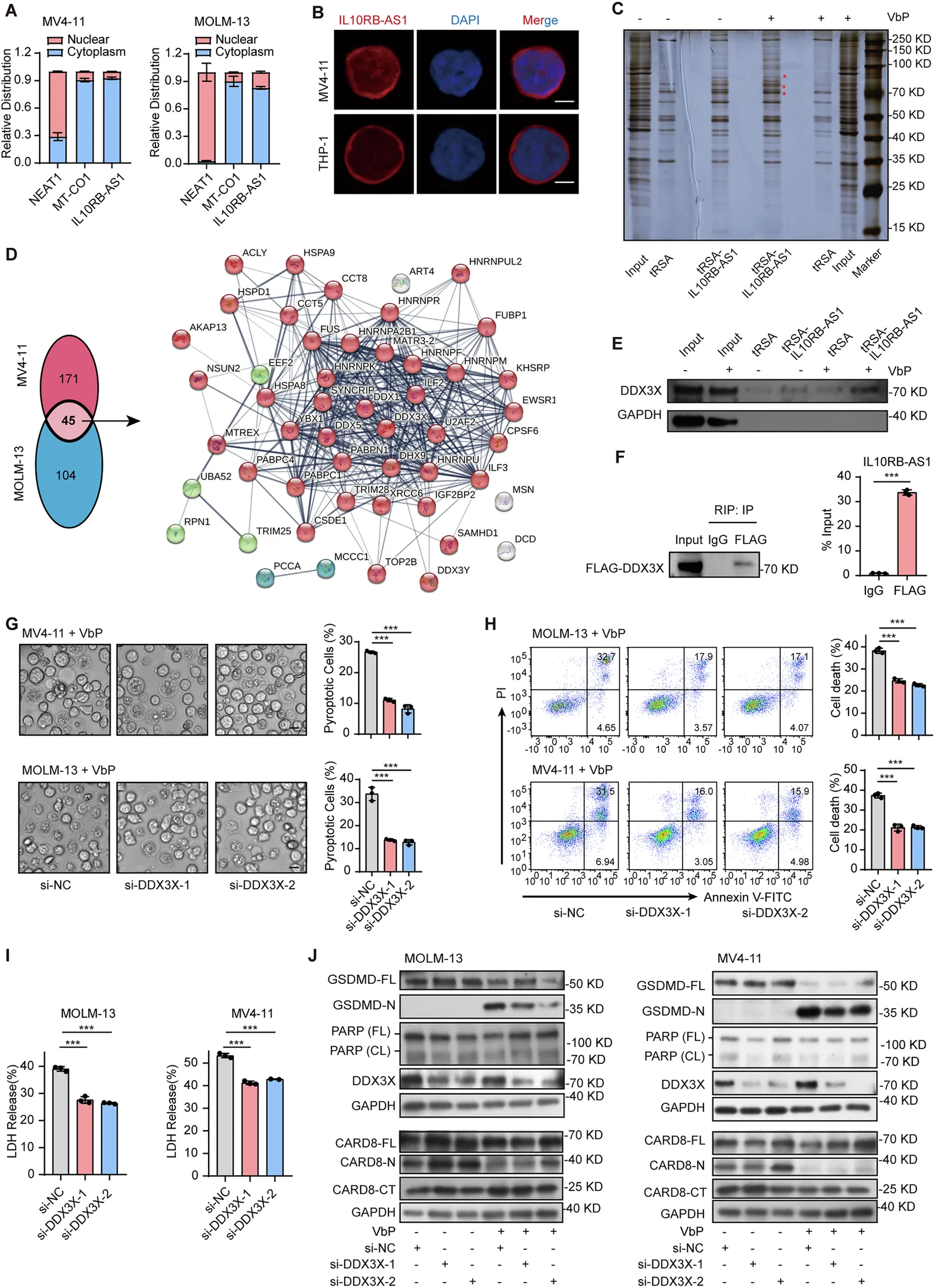
lncRNA IL10RB-AS1 interacts with DDX3X to activate pyroptosis in AML cells. **A** Subcellular localization of lncRNA IL10RB-AS1 by nuclear-cytoplasmic fractionation. NEAT1 acts as the nuclear marker and MT-CO1 acts as the cytoplasmic marker. **B** RNA fluorescence in situ hybridization (FISH) confirms cytoplasmic enrichment of lncRNA IL10RB-AS1 (red signal). Nuclei are counterstained with DAPI (blue; scale bar: 5 µm). **C** Silver staining showed IL10RB-AS1-interacting proteins enriched by tRSA-IL10RB-AS1 pulldown assay under VbP treatment. **D** The Venn diagram and network illustrating the results of mass spectrometry analysis on differential bands from the RNA pulldown assay in MV4-11 and MOLM-13 cells. **E** Validation of IL10RB-AS1-DDX3X interaction by RNA pulldown coupled with Western blot. GAPDH as negative control. **F** RIP assay using anti-IgG or anti-FLAG followed by quantitative real-time PCR analysis. The percentage of RIP-enriched IL10RB-AS1 relative to the input value. Morphological quantification (**G**) and Flow cytometry analysis (**H**) demonstrates the attenuated VbP-induced pyroptosis in DDX3X downregulated AML cells. **I** LDH release from AML cells after electrotransfection with si-NC or si-DDX3X-1/2 and treatment with 5 µM VbP. Data are normalized to total LDH content. **J** Immunoblots of GSDMD, PARP, DDX3X, and CRAD8 in the DDX3X-silenced AML cells treated with or without 5 µM VbP for 24 hours, showing the protein level of full-length and cleaved GSDMD, PARP, and proteolytic level of CARD8. GAPDH serves as control. FL: full-length, N: N-terminal, CT: C-terminal domain, CL: cleaved. Values are mean ± SD (*n* = 3), one-way ANOVA in **G**–**I**, and unpaired two-tailed Student’s *t*-test in **F**. *** *P* < 0.001.

We next endeared to characterize the mechanism of IL10RB-AS1 regulating the nucleation of CARD8-CT and pyroptosis. We first investigated the subcellular localization of this lncRNA, and the result showed that IL10RB-AS1 was mainly cytoplasm-localization (Figs. 4A, B and S5A). It has been reported that lncRNAs can function in regulatory mechanisms through *cis* or *trans* actions [42]. The results showed that manipulation of IL10RB-AS1 expression did not alter the expression of its adjacent genes, IL10RB and interferon alpha/beta receptor 1 (IFNAR), the latter being an effector of the interferon response (Fig. S5B, C). These data suggest that IL10RB-AS1 does not modulate the inflammatory pathway through direct cis-regulation of its genomic neighbors. We next attempted to identify the proteins that interact with IL10RB-AS1 in cytoplasm. IL10RB-AS1 fused with tRSA to conduct RNA pulldown assays with cell lysis from the MOLM-13 or MV4-11 treated with or without VbP, followed by SDS-PAGE, silver staining, and mass spectrometry (MS). tRSA alone was used as a control (Fig. 4C and S6A). The identified proteins are shown in Table S2. Gene Ontology (GO) analysis showed that the interactome of IL10RB-AS1 was most enriched in “RNA binding”-related categories (Fig. 4D).

Among the proteins potentially interacting with IL10RB-AS1, DExD/H-box (DDX/DHX) helicases (DHX9) and DDX3X attracted our interest. We first investigated whether DHX9, which displayed strong binding scores in the interactome of IL10RB-AS1, involves in the IL10RB-AS1 regulation in AML cells. Previous studies have revealed that DHX9 primarily uses its N-terminal double-stranded RNA binding domain (dsRBD) to bind dsRNA, and its sequence binding specificity is weak [43, 44]. RNA pulldown assay showed that DHX9 binds to both IL10RB-AS1 and the negative control (Fig. S6B), indicating that its function may not be exclusively associated with IL10RB-AS1-mediated regulation. Indeed, knocking down DHX9 cannot affect the VbP-induced the CARD8 inflammasome-mediated pyroptosis (Fig. S6C–E). These data suggest that DHX9 is weakly involved in the IL10RB-AS1-CARD8 regulatory axis during VbP-induced pyroptosis, at least in AML cells.

In a current study, DDX3X was reported to interact with NLRP3 to drive NLRP3 inflammasome activation and promote pyroptosis in macrophages [45]. However, in AML cells, NLRP3 showed limited influence on pyroptosis and cytotoxicity regardless of VbP treatment (Fig. S6F). Thus, we speculated that IL10RB-AS1 might activate CARD8 not NLRP3 inflammasome-mediated pyroptosis via interacting with DDX3X in AML cells. To test this hypothesis, we first validated the interaction between IL10RB-AS1 and DDX3X. RNA pull-down assays followed by western blotting revealed a strong association between IL10RB-AS1 and DDX3X (Figs. 4E and S6G). The specificity of this interaction was further confirmed by RNA immunoprecipitation (RIP) using both FLAG-DDX3X in 293T and endogenous DDX3X in AML cells (Figs. 4F and S6H–I), supporting the feasibility of an IL10RB-AS1–DDX3X complex.

To determine whether the effect of IL10RB-AS1 on CARD8 inflammasome-mediated pyroptosis is associated with DDX3X, we examined the role of DDX3X in DPP8/9 inhibitor-induced pyroptosis. The results showed that, similar to the effects of IL10RB-AS1 knockdown, silencing of DDX3X significantly attenuated the VbP-induced pyroptosis across all tested AML cell lines, including decreased number of pyroptosis cells, inhibiting cell death, and reducing the release of LDH (Fig. 4G–I and S6J–L). Furthermore, DDX3X knockdown inhibited VbP-induced GSDMD cleavage but did not affect CARD8 cleavage (Fig. 4J). Together, these findings highlight a crucial role for DDX3X in DPP8/9 inhibitor-induced pyroptosis. Moreover, IL10RB-AS1 regulates CARD8 inflammasome-mediated pyroptosis, at least partially, through its interaction with DDX3X.

### DDX3X interacts with CARD8 to govern the nucleation of the CARD8 inflammasome and pyroptosis in AML cells

**Fig. 5:**
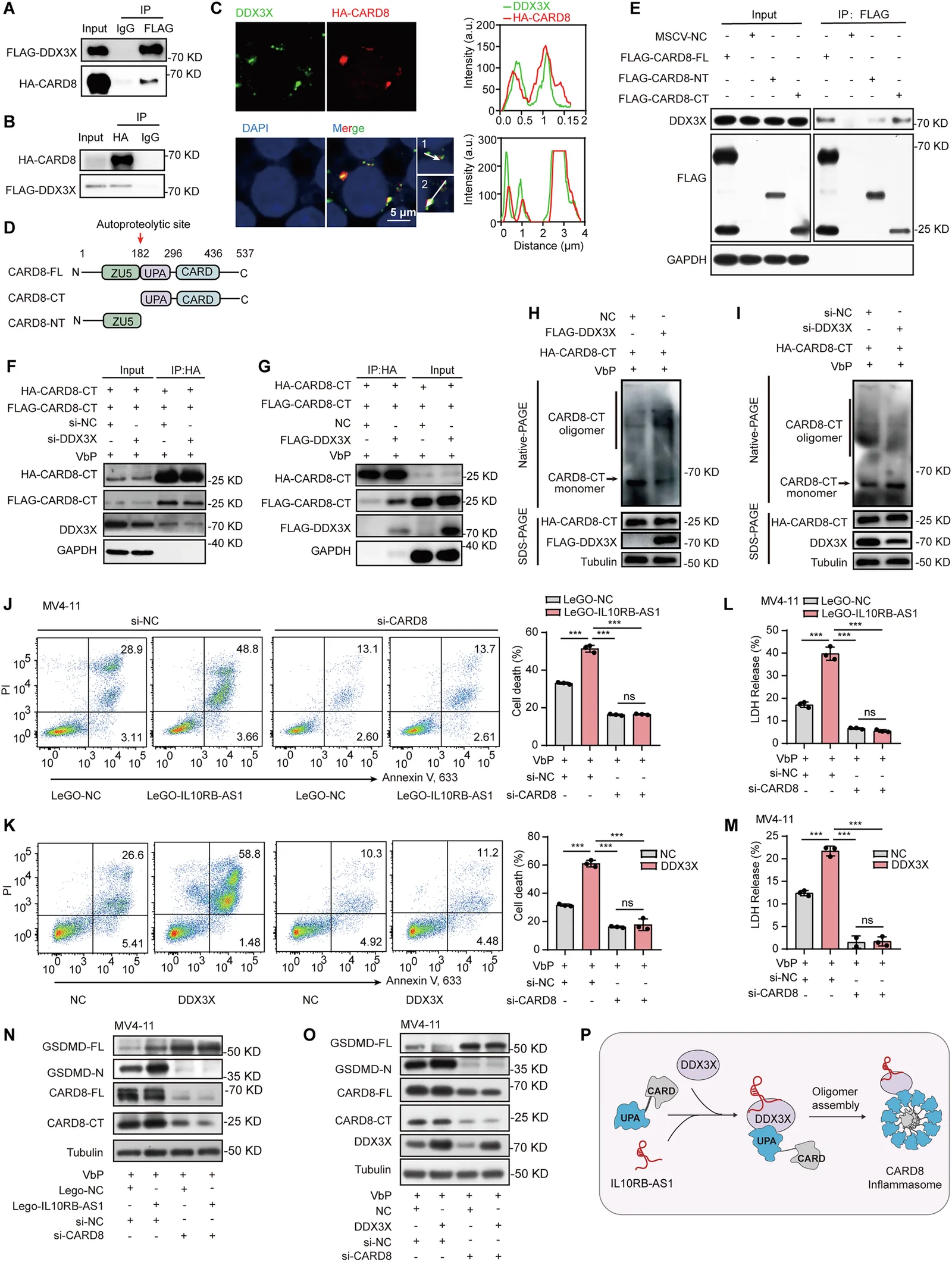
DDX3X interacts with CARD8 and regulate its nucleating, thereby activating pyroptosis. **A**, **B** Co-immunoprecipitation (Co-IP) confirms the interaction between expressed FLAG-DDX3X and HA-CARD8 from HEK293T cell lysates. **C** Immunofluorescence staining reveals co-localization of endogenous DDX3X (green) and exogenously overexpressed HA-tagged CARD8 (red) in the cytoplasm (scale bar: 5 μm). **D** Schematic diagram of the CARD8 protein. **E** Immunoblot analysis showing immunoprecipitation of DDX3X with FLAG-tagged CARD8 constructs. Immunoprecipitation of CARD8-C-terminal interactions in DDX3X-silencing (**F**) or -overexpressing (**G**) cells. Cells were treated with 5 µM VbP for 24 hours. CARD8-CT oligomerization analyzed by native gel under DDX3X overexpression (**H**) or knockdown (**I**). Black arrows, monomeric species. Flow cytometric analysis of cell death (PI+ / Annexin V+ cells) in the IL10RB-AS1 overexpression (**J**) or DDX3X overexpression **(K**) AML cells after electrotransfection with si-NC or si-CARD8 and treatment with 5 µM VbP. LDH release from the IL10RB-AS1 overexpression (**L**) or DDX3X overexpression (**M**) AML cells after electrotransfection with si-NC or si-CARD8 and treatment with 5 µM VbP. Immunoblots of GSDMD and CARD8 in the IL10RB-AS1 overexpression (**N**) or DDX3X overexpression (**O**) AML cells after electrotransfection with si-NC or si-CARD8 and treatment with 5 µM VbP. Tubulin was used as the control. **P** A working model depicting IL10RB-AS1 binding to DDX3X facilitates CARD8 inflammasome assembly. FL: full-length, N: N-terminal, CT: C-terminal domain, CL: cleaved. Values are mean ± SD (*n* = 3), one-way ANOVA in **J**–**M**. ns not significant; ***P < 0.001.

We further studied how DDX3X regulates CARD8 inflammasome-mediated pyroptosis. FLAG-DDX3X and HA-CARD8 were first expressed in HEK293T cells, and CoIP assays showed that FLAG-DDX3X interacted with HA-CARD8 under VbP treatment (Fig. 5A, B). The endogenous interactions of DDX3X and CARD8 were further verified in AML cells (Fig. S6M, N). Subsequently, an immunofluorescence experiment also showed that DDX3X indeed colocalized with HA-CARD8 in the cytoplasm (Fig. 5C), indicating that DDX3X could feasibly interact with CARD8.

CARD8 undergoes the autoproteolysis between the ZU5 and UPA subdomains within its FIIND domain [14, 23]. To identify which region of CARD8 binds to DDX3X, we employed a series of truncated, FLAG-tagged CARD8 fragments (Fig. 5D). Co-immunoprecipitation assays revealed that DDX3X primarily interacts with the CARD8-CT (Fig. 5E). In contrast, DHX9 could not interacts with CARD8-CT (Fig. S6O). These data indicate that DDX3X, not DHX9, regulates DPP8/9 inhibitor-mediated pyroptosis via binding to activating form of CARD8, CARD8-CT. We next investigate whether DDX3X-CARD8-CT interaction can regulate the CARD8-CT oligomerization. As expected, DDX3X knockdown severely impaired the interaction between Flag-CARD8-CT and HA-CARD8-CT, while DDX3X overexpression enhanced this interaction (Fig. 5F, G). Moreover, in a Native-PAGE assay, DDX3X overexpression significantly promoted the CARD8-CT oligomerization (Fig. 5H). Conversely, its knockdown suppressed the formation of these higher-order oligomers (Fig. 5I). These results have suggested a pivotal role for DDX3X in CARD8-CT oligomer assembly and CARD8 inflammasome activity, a function that parallels its binding partner IL10RB-AS1.

We next sought to determinate whether the IL10RB-AS1 / DDX3X-regulated AML cell pyroptosis depend on inflammasome activity. To test this hypothesis, we performed a series of rescue experiments. As expected, overexpression of either IL10RB-AS1 or DDX3X slightly affected VbP-mediated pyroptosis—including cell death, LDH release, and GSDMD cleavage in CARD8-knockdown cells (Fig. 5J–O). Collectively, these results suggest that IL10RB-AS1 / DDX3X may facilitate the nucleation of the CARD8 inflammasome and linker the changes of cellular homeostasis into the activation of CARD8-mediated pyroptosis in AML cells (Fig. 5P).

### IL10RB-AS1 orchestrates DDX3X / CARD8 complex in AML cells

**Fig. 6:**
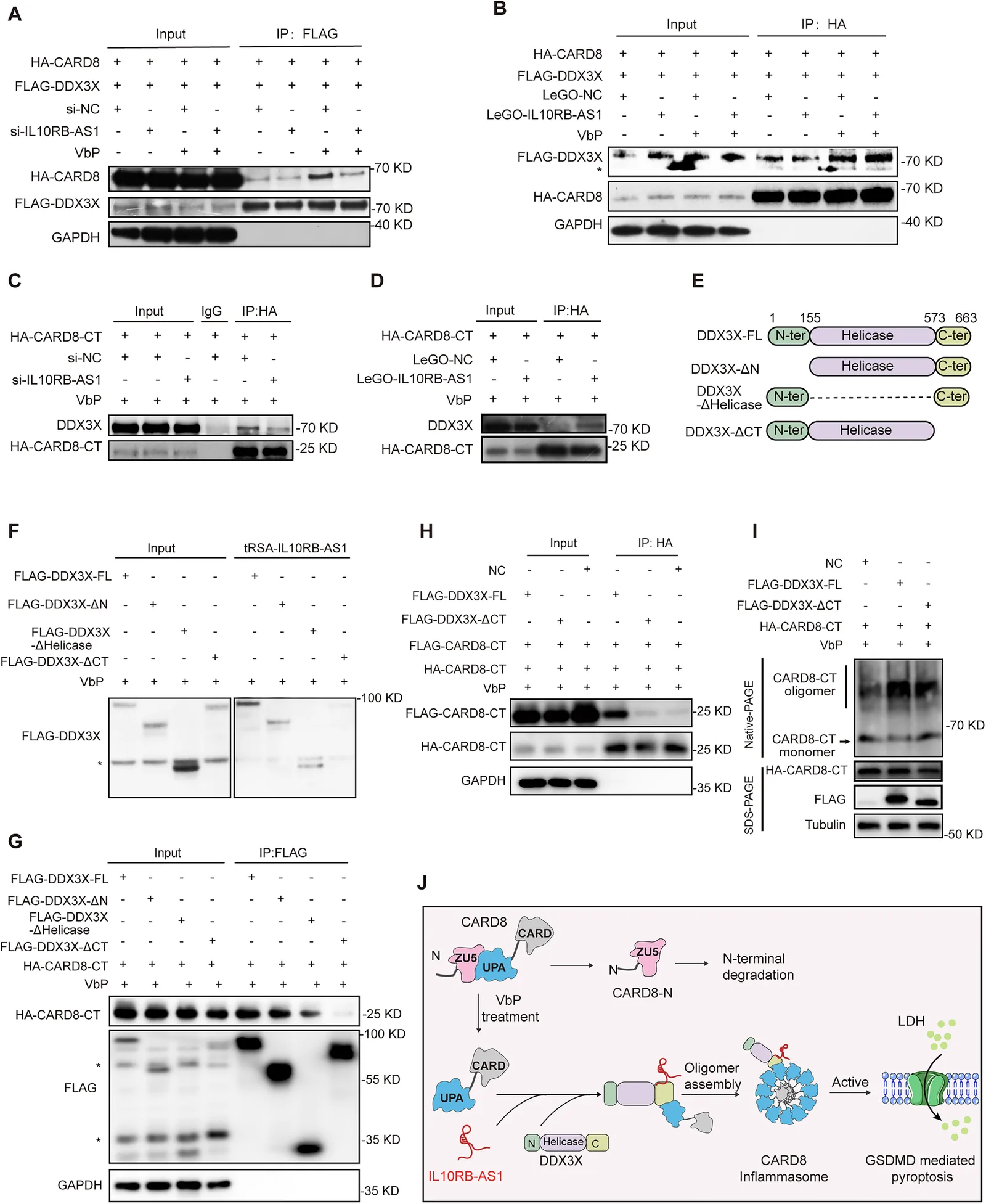
lncRNA IL10RB-AS1 facilitates DDX3X / CARD8 complex assembly through domain-specific interactions. Immunoblot analysis of co-immunoprecipitated HA-CARD8 and FLAG-DDX3X upon knockdown (**A**) or overexpression (**B**) of IL10RB-AS1. GAPDH serves as control. Immunoblots showing the interacts between HA-CARD8-CT and DDX3X under downregulation (**C**) or upregulation (**D**) of IL10RB-AS1. **E** Schematic for DDX3X -domain-deletion mutants. Δ, deletion of the indicated domain. **F** RNA pulldown showing the IL10RB-AS1 interacts with FLAG-tagged DDX3X domain-deletion mutants. *, indicates the non-specific band. FLAG-tagged DDX3X domain deletion mutants includes: Full-length DDX3X (FLAG-DDX3X-FL), DDX3X with N-teminal deletion (FLAG-DDX3X-ΔN), DDX3X with helicase deletion (FLAG-DDX3X-ΔHelicase), DDX3X with C-teminal deletion (FLAG-DDX3X-ΔC). **G** HA-CARD8-CT Co-IP with and FLAG-tagged DDX3X domain-deletion mutants. GAPDH serves as control. *, indicates the non-specific band. **H** Immunoblot detection of HA-CARD8-CT and FLAG-CARD8-CT interaction following FLAG-DDX3X-FL and FLAG-DDX3X-ΔC expression in HEK293T cells under VbP treatment. GAPDH serves as control. **I** CARD8-CT oligomerization analyzed by native gel under overexpression of FLAG-DDX3X-FL or FLAG-DDX3X-ΔC. Black arrow, monomeric species. **J** Schematic diagram illustrating the regulatory role of IL10RB-AS1 in pyroptosis: IL10RB-AS1 binding to DDX3X-CT promotes CARD8 inflammasome assembly, which activates the GSDMD-mediated pyroptosis.

We asked whether IL10RB-AS1 orchestrates DDX3X / CARD8 pyroptosis in AML cells. Pulldown assays showed that IL10RB-AS1 binds slightly to CARD8-CT but moderately to DDX3X, indicating a possible that IL10RB-AS1 may regulate CARD8 inflammasome nucleation by directly binding to DDX3X, thereby enhancing the interaction between DDX3X and CARD8-CT (Figs. 4E and S6P). To this end, we employed Co-IP assays in cells engineered for IL10RB-AS1 knockdown or overexpression. The results showed that IL10RB-AS1 silencing can significantly diminish the interaction of CARD8 with DDX3X in the cells under VbP treatment (Fig. 6A). Reciprocally, the bind of DDX3X to CARD8 was improved in IL10RB-AS1 overexpression cells under VbP treatment (Fig. 6B), indicating that the interactions between CARD8 and DDX3X are regulated by IL10RB-AS1 in DPP8/9 inhibitor-induced pyroptosis. Further Co-IP assays had been observed that IL10RB-AS1 regulated the bind of DDX3X to the CARD8-CT (Fig. 6C, D), suggesting that IL10RB-AS1 can activate CARD8 inflammasome by administrating the interaction of DDX3X to activated form of CARD8 (CARD8-CT) nucleation under DPP8/9 inhibitor treatment.

We next investigated the functional domain of DDX3X responsible for its interaction with IL10RB-AS1 and CARD8-CT by use of domain-deletion mutants (Fig. 6E). Deleting C-terminal domain (DDX3X-ΔCT) significantly impaired the IL10RB-AS1-DDX3X interaction (Fig. 6F), suggesting that the C-terminal domain of DDX3X is pivotal for IL10RB-AS1 binding. Interestingly, deleting the C-terminal domain of DDX3X also significantly impaired the interaction of DDX3X with CARD8-CT in the cell exposed to VbP (Fig. 6G). This finding may indicate that C-terminal domain of DDX3X may confer the function in regulation CARD8-CT oligomer assembly. As expected, in contrast to full-length DDX3X, overexpression of DDX3X-ΔCT did not affect CARD8-CT oligomerization (Fig. 6H, I). Thus, the C-terminal domain of DDX3X is required for its interaction with both IL10RB-AS1 and CARD8-CT, and thereby facilitates the formation of higher-order CARD8-CT oligomers.

Collectively, it could be proposed that IL10RB-AS1 is a pivotal regulator for DPP8/9 inhibitor-induced pyroptosis and functions as a “steerer” by binding C-terminal domain of DDX3X to enhance the interaction of DDX3X and CARD8-CT, thereby activating CARD8 inflammasome-nucleating and pyroptosis in AML cells (Fig. 6J).

### IL10RB-AS1 promotes the DPP8/9 inhibitor -mediated pyroptosis of AML cells in vivo

**Fig. 7:**
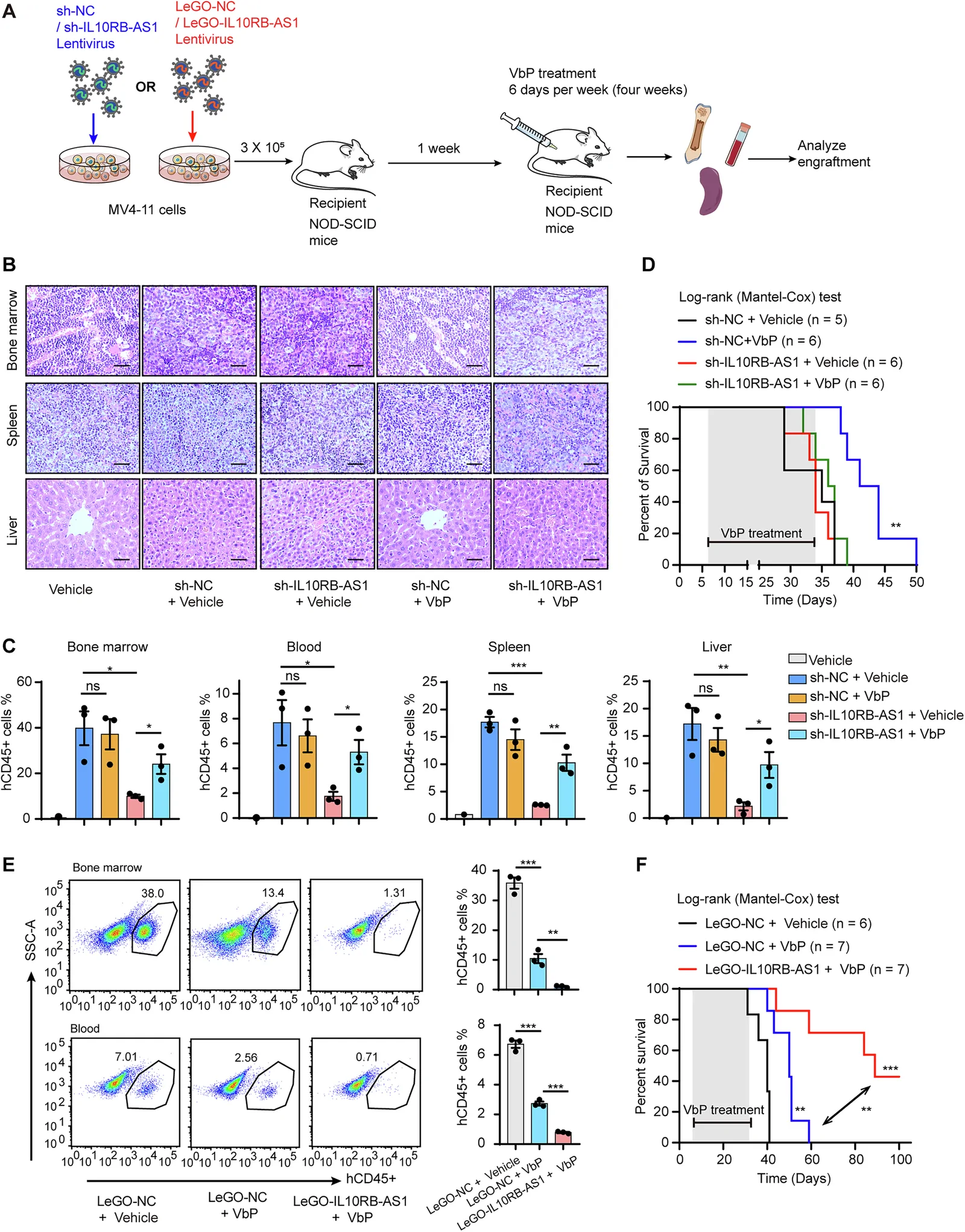
IL10RB-AS1 confers the VbP-mediated pyroptosis of AML in vivo. **A** Schematic diagram of xenograft formation in NOD / SCID mice. The mice intravenously injected sh-NC / sh-IL10RB-AS1 or LeGO-NC / LeGO-IL10RB-AS1 MV4-11 cells, followed by intraperitoneal VbP (1 mg/kg per dose per mouse) inoculation for six consecutive days per week. **B** Representative images of Hematoxylin and eosin (H&E) staining reveals leukemic infiltration in bone marrow, spleen, and liver of sh-L10RB-AS1 mice compared to sh-NC controls, under vehicle or VbP treatment. Mice treated with vehicle were the negative control. Scale bars: 50 µm. **C** Statistical data of flow cytometry analyses of leukemia cells (hCD45+) in Bone Marrow, peripheral blood, spleen, and liver of NOD / SCID mice injected vehicle, sh-NC, and sh-IL10RB-AS1 with vehicle or VbP treatment. hCD45+ indicates the proliferation status of human MV4-11 cells in mice. **D** Kaplan–Meier survival analysis showed that VbP treatment prolongs survival in sh-NC mice but fails to rescue the shortened survival in sh-IL10RB-AS1 mice (log-rank (Mantel-Cox) test: sh-NC + VbP vs. sh-IL10RB-AS1 + VbP). **E** Representative flow cytometry graphs(left) and histogram plots (right) showing the blasts in organ samples from mice injected with LeGO-NC and LeGO-IL10RB-AS1 cells following VbP (1 mg/kg per dose per mouse) treatment or not. **F** Kaplan–Meier survival curves showing the survival of mice implanted with LeGO-NC or LeGO-IL10RB-AS1-transfected MV4-11 cells treated with vehicle or VbP. Log-rank (Mantel-Cox) test was used to analyze the LeGO-NC + Vehicle vs. LeGO-NC + VbP, LeGO-NC + Vehicle vs. LeGO-IL10RB-AS1 + VbP, and LeGO-NC + VbP vs. LeGO-IL10RB-AS1 + VbP. Values are mean ± SEM (*n* = 3), one-way ANOVA in **C**, **D**. ns not significant; *P < 0.05; **P < 0.01; ***P < 0.001.

We finally evaluate the role of IL10RB-AS1 in AML in vivo. The xenograft mouse model was first established by intravenously injecting MV4-11 cells transfected with sh-IL10RB-AS1 and control (named sh-NC) into the tail vein of NOD / SCID mice, and then we initiated the intraperitoneal injection of the animals with the DPP8/9 inhibitor on day 7 after transplantation, before they succumbed to leukemia (Figs. 7A and S7A). Intraperitoneal injection with 1 mg/kg of VbP or vehicle was performed six consecutive days per week (four weeks). Four treatments by injection were administered—sh-NC cells, sh-IL10RB-AS1 cells, sh-NC + VbP, and sh-IL10RB-AS1+ VbP. At day 35, we randomly killed three mice in each group and investigated the infiltration degree of MV4-11 cells. As shown in hematoxylin and eosin (H&E) staining assays (Fig. 7B), VbP treatment, respectively, led to decreased infiltration ability for AML cells and less liver injury in the tested mice, while, this anti-leukemic effect of VbP treatment was inhibited by IL10RB-AS1 knockdown. The compromised infiltration ability of VbP treatment was consistent with the alleviated tumor burden as testified by the declined percentages of hCD45+ cell populations in the bone marrow, blood, and liver from tested mice, which was consistent with in vitro results (Figs. 7C and S7B). However, the percentages of hCD45+ cells were also dramatically increased in the group of sh-IL10RB-AS1 + VbP compared to those in VbP group, suggesting that IL10RB-AS1 is necessary for VbP-induced cytotoxicity of AML (Figs. 7C and S7B). Notably, the mice in the VbP treatment survived longer than those in other groups; however, sh-IL10RB-AS1 can strikingly limit the effect of VbP treatment (Fig. 7D).

To further assess the potential application value, we subsequently performed another xenograft mouse experiment. In brief, this model was established by injecting MV4-11 cells transfected stably with either LeGO-IL10RB-AS1 or the control (LeGO-NC) into the tail vein of NOD / SCID mice. This experimental procedure is also depicted in Fig. 7A. Importantly, LeGO-IL10RB-AS1 can dramatically improve the VbP treatment to AML, as evidenced by declined percentages of AML cell populations in the bone marrow and blood from tested mice (Fig. 7E). Combining LeGO-IL10RB-AS1 upregulation with VbP treatment significantly prolonged the lifespan of the AML mice (Fig. 7F). Overall, we found that IL10RB-AS1 was a key factor to manage the cytotoxicity of VbP treatment, potential as a promising target in AML therapy.

Taken together, our findings demonstrate that IL10RB-AS1 acts as a novel regulator by interacting with DDX3X to modulate CARD8-CT oligomerization and nucleation, thereby inducing CARD8-mediated pro-caspase-1 pyroptosis in AML cells. We first unmasked that DDX3X plays a crucial role in CARD8 inflammasome-mediated pyroptosis. The C-terminal domain of DDX3X was identified to interact with the C-terminal of CARD8, and IL10RB-AS1 enhances the DDX3X-CARD8 interaction to promote activating CARD8 inflammasome in AML cells (Fig. 6J). This finding not only uncovered a previously unknown mechanism of lncRNA orchestrating the nucleation of CARD8 inflammasome and its-mediated pyroptosis, but also provides new opportunities for DPP8/9 inhibitors in hematopoietic malignancies with highly expressed IL10RB-AS1, and even elevating IL10RB-AS1 levels in cancer cells could be a promising strategy for DPP8/9 inhibitor treatment.

## Discussion

Increasing evidences have shown that pyroptosis is broadly implicated in both disease pathogenesis and therapeutic strategies [8, 11, 12, 46, 47]. The process of multiprotein complexes order-triggered pyroptosis and the diverse activating mechanisms of inflammasome-nucleating proteins remain to be fully elucidated, especially in disease with certain context. Specifically, CARD8 is highly expressed in hematopoietic cells, and its inflammasome triggers pro-caspase-1-dependent pyroptosis in AML cells upon DPP8/9 inhibition, indicating it as a potential therapeutic target in AML [14–18]. Recent studies expanded our understanding of process in CARD8-mediated pyroptosis; however, the molecular details of CARD8-CT oligomeric assembly and nucleation, as well as its function in AML, remain poorly characterized. In this study, we first identified a lncRNA IL10RB-AS1 that candidates as a novel regulator that administrates the CARD8-CT oligomer assembly and promotes CARD8 inflammasome mediated-pyroptosis in AML cells. Importantly, we uncovered that IL10RB-AS1 enhances the DDX3X-CARD8 interaction to promote oligomerization and nucleation. This is the first report revealing that a lncRNA can orchestrate CARD8 inflammasome activation, providing a potential avenue of innovative strategies in how to treat hematopoietic malignancies with highly expressed IL10RB-AS1 contexts.

CARD8 is a non-canonical pattern recognition receptor that nucleates the inflammasome in response to host-associated molecular patterns (HAMPs), mediating ASC-independent pyroptosis [14, 16–18, 48, 49]. It is highly expressed in hematopoietic cells, making them an ideal model for studying CARD8-mediated pyroptosis [17, 41]. Previous studies have shown that DPP8/9 inhibitors and pathogenic HIV infection can activate CARD8 inflammasome, leading to pyroptotic cell death [40, 48, 49]. Recent studies have exhibited the pathways of CARD8 / pro-caspase-1 / GSDMD in pyroptosis; however, the factors that link alterations in cellular homeostasis to the activation of CARD8-mediated pyroptosis remain poorly understood. In the current study, we provide substantial experimental evidence demonstrating that IL10RB-AS1, in association with DDX3X, can activate CARD8-mediated pyroptosis in AML cells under VbP treatment. This finding may propose IL10RB-AS1 potential as regulator that can linker the changes of cellular homeostasis into the activation of CARD8-mediated pyroptosis.

Several studies have demonstrated some pathways involved in CARD8 NT degradation and the release of CARD8-CT [14, 23–25]. For instance, the disordered region of N-terminus of CARD8 may facilitate proteasomal degradation, leading to the release of its C-terminal fragment and subsequent activation of CASP1 [24]. However, the underlying mechanisms governing CARD8-CT oligomer assembly and nucleation remain largely unknown. In this study, we found that IL10RB-AS1 does not affect the proteasomal degradation of the N-terminal fragments of CARD8, but may regulate the CARD8-CT oligomer assembly and nucleation. Specifically, IL10RB-AS1 recruits DDX3X to the C-terminal fragment CARD8, promoting its oligomerization and nucleation, thereby inducing pyroptosis in AML cells. These findings enhance our understanding of the complex mechanisms underlying CARD8 inflammasome nucleation and pyroptotic activation. Furthermore, our study is the first to report that a lncRNA can orchestrate CARD8-CT oligomeric assembly and nucleation, leading to CARD8 inflammasome-mediated pyroptosis in AML cells.

Previous studies have exhibited that inducing pyroptosis represents a promising strategy for cancer treatment, both through the release of anti-tumor inflammatory factors and the induction of lytic cell death [11, 12, 46, 47, 50, 51]. A recent study showed that DPP8/9 inhibitors, such as VbP, induce the proteasome-mediated degradation of the CARD8-N, thereby releasing free CT fragments and activate the CARD8 inflammasome, thereby promoting the lytic cell death in AML, a hematopoietic malignancy with a very poor prognosis, with limited effects of specific therapies, and frequent treatment resistance [18]. Rao et al. also design a small molecule called CQ31 that inhibits the M24B aminopeptidases prolidase (PEPD) and Xaa-Pro aminopeptidase 1 (XPNPEP1), inhibiting DPP8/9 and thereby only activating CARD8 inflammasome [16]. These studies may propose a therapeutic strategy for AML of using DPP8/9 inhibitors. In the present study, we identify that cell sensitive to VbP exhibit high expression of IL10RB-AS1. Downregulation of IL10RB-AS1 significantly attenuated the DPP8/9 inhibitor-induced pyroptosis. It is possible that IL10RB-AS1 may be potential as a predictor of DPP8/9 inhibitor sensitivity. Interestingly, upregulation of IL10RB-AS1 can improve the VbP-induced pyroptosis. These findings may represent an excellent strategy to treat cancers using DPP8/9 inhibitors via elevating IL10RB-AS1 expression.

DDX3X is universally expressed in eukaryotes and plays critical roles in RNA metabolism, participating in cell signal transduction and stress granule regulation, which are essential for maintaining cellular homeostasis [45, 52–55]. Previous evidence indicates that DDX3X is involved in both pro-death and pro-survival pathways, leading to an ongoing controversy persists as to whether regarding its identity as a suppressor or activator gene [45, 52, 56]. An interesting study has mentioned that DDX3X can act as a live-or-die checkpoint in stressed cells by regulating NLRP3 inflammasome in bone-marrow-derived macrophages [45]. Specifically, upon lipopolysaccharide (LPS) stimulation, DDX3X interacts with NLRP3 to promote inflammasome activation [45]. This suggests that macrophages utilize DDX3X to sense stress signals and switch between pro-survival stress granules and pyroptotic ASC specks [45, 57]. Notably, in AML cells, we found that DDX3X interacted with CARD8 rather than NLRP3, leading to activation of CARD8 inflammasome-mediated pyroptosis. Functional studies further confirmed that CARD8, but not NLRP3, influences VbP-induced pyroptosis and cytotoxicity in AML cells. These observations suggest that DDX3X promotes the ASC-dependent pyroptosis through interaction with NLRP3 in response to pathogen-associated molecular patterns (PAMPs), such as LPS, in healthy macrophages. In contrast, DDX3X-CRAD8 axis exerts the CARD8 inflammasome-mediated pyroptosis in hematopoietic malignancies, such as AML cells. Interestingly, the binding site for NLRP3 localizes to the helicase domain of DDX3X, whereas CARD8 primarily binds to the C-terminal domain. This domain-specific interaction may reflect distinct regulatory mechanisms. Thus, our work, together with recent findings, underscores that the functional roles and regulatory mechanisms of DDX3X are both spatiotemporal and context-dependent.

In conclusion, our results show that IL10RB-AS1 orchestrates the oligomerization and nucleation of CARD8-CT in response to changes in cellular homeostasis and activates CARD8-mediated pyroptosis in AML. Specifically, IL10RB-AS1 recruits DDX3X and enhances the DDX3X-CARD8 interaction to facilitate CARD8-CT nucleation, thereby activating CARD8 inflammasome and inducing ASC-independent pyroptosis. Notably, we identified that IL10RB-AS1 is highly expressed across multiple AML subtypes, suggesting its potential use as a predictive biomarker for sensitivity to DPP8/9 inhibitors. These findings not only highlight the role of lncRNA in orchestrating CARD8 inflammasome nucleation, but also open new therapeutic avenues for activating CARD8-mediated pyroptosis in hematopoietic malignancies with highly expressed IL10RB-AS1. Moreover, increasing IL10RB-AS1 levels in cancer cells could be a promising strategy for pyroptosis-based therapy.

## Methods

### Patients

All patient samples were obtained at the time of diagnosis or relapse and with informed consent from the First Affiliated Hospital of Sun Yat-sen University. Sample collection was approved by the Hospital’s Protection of Human Subjects Committee. The detailed clinicopathological characteristics of the patients were summarized in Table S1.

### Cell culture

The human leukemia cell lines MOLM-13 (DSMZ, RRID: CVCL_2119), MV4-11 (ATCC, RRID: CVCL_0064), HL-60 (ATCC, RRID: CVCL_0002), THP-1 (ATCC, RRID: CVCL_0006), U937 (ATCC, RRID: CVCL_0007), and the human embryonic kidney cell line HEK293T (ATCC, RRID: CVCL_0045) were used. MOLM-13, THP-1, and U937 were cultured in RPMI-1640 medium (Hyclone, USA) supplemented with 10 % fetal bovine serum (Gibco, Cat# A5256701, ThermoFisher). HL-60 cells were cultured in RPMI-1640 medium supplemented with 20 % FBS. MV4-11 cells were cultured in IMDM medium (Gibco, Cat# 12200036, ThermoFisher) supplemented with 10 % FBS; HEK293T cells were cultured in DMEM medium (Gibco, Cat# 10313021, ThermoFisher) supplemented with 10 % FBS. All cells were maintained at 37 °C in a 5 % CO₂ atmosphere.

The purchase dates for the cell lines were missing. We have confirmed that all of the cell lines used in this study were detected by Mycolor One-Step Mycoplasma Detector (Vazyme, Cat# D201-01) and were contamination free. The Research Resource lentifiers (RRlDs) of some materials were missing. We stated that the cell lines used are well-established models in the field, and the missing information mentioned above does not impact the study’s conclusions.

### Plasmid construction

shRNA oligonucleotides targeting IL10RB-AS1 were synthesized and cloned into the pGreenPuro eukaryotic expression vector, with sh-NC serving as the negative control. Simultaneously, full-length IL10RB-AS1 amplified from MV4-11 cDNA was subcloned into the LeGO-puro vector. Wild-type CARD8 full length (CARD8-FL), truncated mutants (CARD8-N [1-296 aa], CARD8-CT [297-537 aa]), full-length DDX3X (DDX3X-FL), and domain-deletion mutants (DDX3X-ΔN [156-662 aa], DDX3X-ΔHelicase [Δ156-573 aa], DDX3X-ΔC [156-573 aa]) were PCR-amplified from MV4-11 cDNA and subsequently cloned into an engineered pCDH vector expressing C-terminal HA or FLAG tag. All primer and shRNA sequences are detailed in Table S3.

### Cell transfection and lentivirus infection

Adherent cells were transfected with overexpression vectors and siRNA using Lipofectamine™ 2000 (Invitrogen, Cat# 11668-019) or Lipo6000™ (Beyotime, Cat# C0526) lipofectamine reagents, strictly adhering to the manufacturers’ protocols. Suspension cells underwent siRNA transfection via electroporation using the Neon™ Transfection System (Invitrogen, Cat# N1096), as specified by the manufacturer. To establish stable cell lines with gene knockdown or overexpression, lentiviral particles were generated by co-transfecting HEK293T cells with the packaging plasmids psPAX2 and pMD2.G, alongside the target gene expression vector. Subsequently, 3 × 10⁵ leukemia cells were collected, resuspended in pre-warmed lentiviral supernatant, and incubated at 37 °C under 5% CO₂ for 24 h. Puromycin selection (1 μg/mL) was applied, followed by harvest of a cell subset for RNA / protein extraction to assess alterations in target gene transcription or protein expression. All siRNA sequences are listed in Table S3.

### RNA isolation and quantitative real-time PCR (RT-PCR)

Cultured cell samples were lysed with RNAiso™ Plus reagent (Takara, Cat# 9109). Total RNA was then extracted according to the manufacturer’s protocol. The purified RNA was dissolved in DEPC-treated water, and its concentration was quantified using a NanoDrop™ 2000 spectrophotometer (ThermoFisher, Cat# ND2000). All RNA samples were stored at −80 °C for subsequent analysis. RNA was reverse transcribed to cDNA using the PrimeScript™ RT Reagent Kit (Takara, Cat# RR047A). Prior to reverse transcription, 500 ng of total RNA per reaction was treated with DNase I to remove genomic DNA contamination. cDNA was generated using random primers. Quantitative PCR amplification was conducted with TB Green™ Premix Ex Taq™ (Takara, Cat# RR420A) on an ABI QuantStudio™ 6 Flex Real-Time PCR System (Applied Biosystems, Cat# 4485692). Thermal cycling conditions followed the manufacturer’s specifications. Gene expression levels were normalized to GAPDH as an endogenous control. Relative quantification was calculated using the comparative 2^−ΔΔCt^ method. Primer sequences were shown in Table S3.

### CCK-8 cell proliferation assay

Cell proliferation was evaluated using the Cell Counting Kit-8 (CCK-8, Dojindo, Japan, CK04). siRNA-transfected cells were seeded in 96-well plates at a density of 2 × 10⁴ cells per well in 100 µL of culture medium. Cell viability was assessed at 0, 24, 48, 72, and 96 hours (h). At each time point, 10 µL CCK-8 reagent was added per well, followed by 3 h incubation at 37 °C under 5 % CO_2_. Subsequently, absorbance was measured at 480 nm and 630 nm using a VICTOR X5™ Multilabel Plate Reader (PerkinElmer, Waltham, USA).

### Flow cytometric analysis

Cell death quantification was performed using an Annexin V-FITC/PI Apoptosis Detection Kit (Dojindo, Japan, Cat# AD10) according to the manufacturer’s protocol. Following centrifugation at 500 × *g* for 3 min, cell pellets were washed with PBS, then resuspended in apoptosis buffer containing Annexin V-FITC and propidium iodide. After thorough resuspension, cells were incubated for 15 minutes (min) at room temperature protected from light. Samples were analyzed using a BD FACSCelesta flow cytometer (BD Biosciences, Cat# FACSCelesta BVR).

### 5′ RACE and 3′ RACE

The 5′- and 3′-ends of IL10RB-AS1 were amplified from MV4-11-derived cDNA using the 5′-Full RACE Kit with TAP (Takara, Cat# D315) and 3′-Full RACE Core Set with PrimeScript™ RTase (Takara, Cat# 6106) following manufacturer protocols. PCR products were cloned into the pEASY-T3 cloning vector (TransGen Biotech, Cat# CT301-01) for Sanger sequencing. All of the primers for RACE experiments are listed in Table S3.

### RNA immunoprecipitation and pull-down assay

RNA immunoprecipitation (RIP) assays were conducted using the Magna RIP™ RNA-Binding Protein Immunoprecipitation Kit (Millipore, Cat# 17-700) according to the manufacturer’s instructions. RNA pull-down assays employed the Pierce Magnetic RNA-Protein Pull-Down Kit (ThermoFisher, Cat# 20164), also following the manufacturer’s protocol [32, 34]. Briefly, the IL10RB-AS1 sequence was amplified by PCR to incorporate T7 promoter and tRSA sequences. The resulting RNA product was synthesized via in vitro transcription using the TranscriptAid T7 High Yield Transcription Kit (ThermoFisher, Cat# K0441) and purified using the GeneJET RNA Purification Kit (ThermoFisher, Cat# K0731). The purified RNA was then folded by gradient cooling from 85 °C to 4 °C. Streptavidin-coated magnetic beads were washed, incubated with the folded RNA in RNA Capture Buffer for 30 min at room temperature, and followed by additional washes. Subsequently, the beads were incubated with cell lysate in Protein-RNA Binding Buffer for 1 h at 4 °C. After extensive washing with Wash Buffer, bound proteins were eluted using Elution Buffer. Eluates were denatured with loading buffer and analyzed by silver staining followed by mass spectrometry (MS) identification and western blotting.

### Co-IP and immunoprecipitation (IP) assay

HEK293T cells transfected with indicated plasmids were incubated for 24 h, followed by 24 h treatment with VbP. Cell pellets were harvested via centrifugation (1000 × *g*, 3 min) and resuspended in co-immunoprecipitation lysis buffer (50 mM Tris-HCl pH 7.4, 150 mM NaCl, 1 mM EDTA, 1 % Triton X-100) containing protease inhibitor cocktail (ThermoFisher, Cat# 78430). Lysis proceeded for 1 h at 4 °C with constant rotation. Cellular debris was removed by centrifugation (16,000 × *g*, 15 min, 4 °C), and the resulting supernatant was incubated overnight at 4 °C with species-matched anti-FLAG (Sigma-Aldrich, Cat# F1804) or anti-HA (Cell Signaling Technology, Cat# 3724) antibodies. Pierce™ Protein A/G Magnetic Beads (ThermoFisher, Cat# 88803) were subsequently added for 2 h incubation. Following three 1-mL washes with wash buffer (50 mM Tris-HCl pH 7.4, 150 mM NaCl, 1 mM EDTA), bead-bound complexes were resuspended with 5× Loading buffer and denatured at 95 °C for 10 min.

### Protein extraction and immunoblotting

Total cellular protein was extracted using RIPA lysis buffer (Beyotime Biotechnology, Cat# P0038) supplemented with protease inhibitors, per manufacturer guidelines. Protein samples were resolved by 10 % SDS-PAGE electrophoresis and subsequently electrotransferred onto 0.45 μm PVDF membranes (Millipore, Cat# IPVH00010). Membranes were blocked with 5 % bovine serum albumin (BSA) for 1 h at room temperature, then incubated overnight at 4 °C with species-specific primary antibodies. Following three washes with Tris-buffered saline containing 0.1% Tween-20 (TBST), membranes were probed with horseradish peroxidase (HRP)-conjugated secondary antibodies for 1 h. Protein bands were ultimately visualized using enhanced chemiluminescence (ECL) detection reagents.

### Animal model

Five-week-old NOD-SCID mice were maintained under pathogen-free conditions at the Laboratory Animal Center of Sun Yat-sen University (Approval No. SYSU-IACUC-2021-B0521), with all procedures following institutional ethical guidelines for animal experiments [58–60]. Mice were randomly divided into three groups (9 mice for each group) and injected via the tail vein with 200 μL of PBS, PBS suspension containing 3 × 10^5^ sh-NC (or LeGO-NC) MV4-11 cells, or sh-IL10RB-AS1(or LeGO-IL10RB-AS1) MV4-11 cells per mouse to establish a leukemia xenograft model. One week post-inoculation, mice from the sh-NC (or LeGO-NC) and sh-IL10RB-AS1(or LeGO-IL10RB-AS1) groups were further randomized to receive daily intraperitoneal injections of 100 μL vehicle (DMSO-containing solvent) or VbP (1 mg/kg) for 6 consecutive days per week. After four weeks of treatment, a subset of xenografted mice was euthanized for analysis. Human cell engraftment (hCD45+ cells) in bone marrow, peripheral blood, and liver was assessed by flow cytometry, while remaining mice were subjected to survival analysis.

### RNA fluorescence in situ hybridization

Subcellular localization of lncRNA IL10RB-AS1 was analyzed by RNA fluorescence in situ hybridization (FISH) in MV4-11 and THP-1 leukemia cells using the RiboTM Fluorescent In Situ Hybridization Kit (RIBOBIO, Cat# P202409240022, China). Briefly, a total of 1 × 10⁶ cells were collected, washed twice with ice-chilled PBS, and fixed in 4% paraformaldehyde (PFA) at room temperature for 10 min. After PBS washes, cells were permeabilized with 0.5 % Triton X-100 in PBS at 4 °C for 5 min. The permeabilization solution was removed, followed by three additional PBS washes. Subsequent hybridization with lncRNA-specific probes was performed strictly according to the manufacturer’s protocol.

### Immunofluorescence

HEK293T cells were seeded in 35-mm glass-bottom culture dishes (MetTek, Cat# P35G-0-14-C), transiently transfected with HA-CARD8 plasmid, and cultured for 24 h prior to 24 h treatment with VbP. After washing with PBS, cells were fixed in 4% paraformaldehyde (PFA) on ice for 10 min, then permeabilized and blocked with immunofluorescence blocking buffer containing 0.5 % Triton X-100 at room temperature for 1 h. After brief rinsing, samples were incubated overnight at 4 °C with rabbit anti-DDX3X (Proteintech, Cat# 67915-1-Ig) and mouse anti-HA primary antibodies (Abmart, Cat# M20003). The next day, dishes were washed thrice with PBS, and subsequently incubated for 1 h at room temperature (protected from light) with secondary antibodies. Following three additional PBS washes, nuclei were counterstained with DAPI, and confocal imaging was performed using a Zeiss LSM880 microscope with Fast Airyscan using 404 nm, 488 nm, and 594 nm laser excitation.

### LDH cytotoxicity assays

Leukemia cell lines (MOLM-13, MV4-11, THP-1, HL-60) were seeded at a density of 3 × 10^4^ cells per well in 96-well plates containing standard growth medium and treated with DMSO or VbP. After predetermined time points, LDH activity was determined using the Cytotoxicity LDH Assay Kit-WST^®^ (Dojindo, Cat# CK12) according to the manufacturer’s protocols.

### Statistical analysis

Statistical analyses were performed using unpaired Student’s *t*-test. Data are presented as mean ± SD or SEM from independent experiments. For comparisons among multiple groups, one-way ANOVA, two-way ANOVA (assuming equal standard deviations between groups), and the Kruskal–Wallis test were applied as appropriate. Multiple comparisons were analyzed using the LSD post-hoc test. Leukemia-free survival was defined as the time from achieving complete remission (CR) to either relapse or death while in remission and was evaluated using the Kaplan–Meier method, with comparisons made by log-rank test. Two-tailed tests were used for all univariate comparisons. Univariate and multivariate analyses of prognostic factors were conducted using Cox proportional hazards regression models. All experiments included at least three biological replicates, as detailed in the figure legends. No blinding was implemented during the study. Statistical significance was defined as follows: *P < 0.05, **P < 0.01, and ***P < 0.001.

## Supplementary information

A clean version of Supplementary Materials

Full and uncropped western blots

## Data Availability

All the study data are included in the article and/or Supplemental Information. Any information required to reanalyze the data reported in this study is available from the corresponding authors (wangwt8@mail.sysu.edu.cn) upon request.
