# Supplementary material for: IL10RB-AS1 promotes pro-caspase-1-dependent pyroptosis via regulating the oligomerization and nucleation of CARD8 C-terminal domain to suppress acute myeloid leukemia
Source: Cell Death Dis. 2026 Jul 31;17(1):909. doi: 10.1038/s41419-026-09129-7 (PMC13714289; doi:10.1038/s41419-026-09129-7)
Supplement: Supplementary file 1 [file 41419_2026_9129_MOESM1_ESM.docx]

**Supporting Information**

**IL10RB-AS1 Promotes Pro-caspase-1-dependent Pyroptosis via Regulating the Oligomerization and Nucleation of CARD8 C-terminal Domain to Suppress Acute Myeloid Leukemia**

**Including:**

- **Supporting Figures and Figure Legends**

Figure S1-S7.

- **Supporting Tables**

Table S1-S4.

**Fig. S1.** **Characterization of lncRNA IL10RB-AS1.**

(**A**) Representative bright-field images showed the pyroptotic cells (characterized by cell swelling and membrane rupture) after 5 µM VbP treatment for 24 hours. Scale bar: 20 µm. (**B**) The schematic of IL10RB-AS1 gene locus. (**C**) Argose gels showing the 3' and 5' RACE analysis of IL10RB-AS1. (**D**) PhyloCSF analysis demonstrates that IL10RB-AS1 lacks protein-coding capacity (*GAPDH* as positive control).

**Fig. S2. Knockdown efficiency of IL10RB-AS1.**

RT-qPCR analysis of IL10RB-AS1 expression levels following siRNA-mediated knockdown in four AML cell lines. Gene expression was normalized to GAPDH. Values are mean ± SD (n = 3), one-way ANOVA was used to analyze the expression differences. *** *P* < 0.001.

**Fig. S3. Functional validation of IL10RB-AS1 in pyroptosis in AML cells.**

(**A**) CCK-8 assay shows no significant effect on proliferation in MOLM-13 and MV4-11 cells upon IL10RB-AS1 knockdown. (**B**) Representative images (left) and flow cytometry quantification (right) of cell death (PI+ / Annexin V+ cells) in AML cells following electrotransfection with si-NC or si-IL10RB-AS1/2 and subsequent treatment with 5 µM VbP. (**C**) LDH release from AML cells after electrotransfection with si-NC or si-IL10RB-AS1-1/2 and treatment with 5 µM VbP. Values are mean ± SD (n = 3), two-way ANOVA in **A**, and one-way ANOVA in **B, C**. ns, not significant; *** *P* < 0.001.

**Fig. S4. IL10RB-AS1 regulates VbP-induced pyroptosis in AML cells.**

(**A**) Immunoblots of PARP or pro-caspase-1 cleavage in AML cells after 10 or 50 µM VbP treatment. GAPDH as the negative control. (**B**) Western blot for the GSDMD in AML cells following treatment with or without 10 µM VX-765, and subsequent treatment with or without 5 µM VbP. GAPDH serves as loading control. FL: full-length, N: N terminus. (**C**) VX-765 rescued the VbP-induced pyroptosis in both MV4-11 and MOLM-13 cell lines. Representative bright-field images (left) and quantitative analysis (right) demonstrate a significant reduction in the number of pyroptotic cells after 10 µM VX-765 treatment followed by 5 µM VbP treatment for 24 hours. N.D.: no canonical pyroptotic cells in the detection. Scale bar:20 μm. (**D**) RT-qPCR showing the levels of GSDMD mRNA and IL10RB-AS1 after IL10RB-AS1 knockdown in AML cell lines. (**E**) Immunoblots showing the cleavage of GSDMD in THP1 and HL-60 cells with IL10RB-AS1 knockdown under 5 µM VbP treatment. ACTIN as the negative control. (**F**) Immunoblots of pro-caspase-1 cleavage in MV4-11 cells following electrotransfection with si-NC or si-IL10RB-AS1 and subsequent treatment with 5 µM VbP. (**G**) RT-qPCR showing the levels of GSDMD mRNA and IL10RB-AS1 in MV4-11 cells stably overexpressing IL10RB-AS1. (**H**) Representative images of Flow cytometry analysis demonstrating cell death percentages in MV4-11 cells stably overexpressing IL10RB-AS1 with or without VbP treatment. FL: full-length, N: N-terminal, CL: cleaved. Values are mean ± SD (n = 3), one-way ANOVA in **D**, and unpaired two-tailed Student’s t-test in **C**, **E**. ns, not significant; * *P* < 0.05; *** *P* < 0.001.

**Fig. S5.** **Indentification of IL10RB-AS1 subcellular localization and its impact on proximal genes.**

(**A**) Subcellular fractionation verifies cytoplasmic localization of IL10RB-AS1 in HL-60 and THP-1 cells. Neat1 as a nuclear marker and MT-CO1 as cytoplasmic marker. (**B and C**) RT-qPCR analysis showing the levels of proximal genes IFNAR and IL10RB after IL10RB-AS1 knockdown (**B**) or overexpression (**C**) in AML cell lines. Values are mean ± SD (n = 3), one-way ANOVA in **B**, and unpaired two-tailed Student’s t-test in **C**. ns, not significant; *** *P* < 0.001.

**Fig. S6. IL10RB-AS1 mediates VbP-induced pyroptosis through binding to DDX3X.**

(**A**) Formaldehyde-denaturing gel electrophoresis of *in vitro* transcribed RNA. tRSA and tRSA-IL10RB-AS1 products were shown. (**B**) IL10RB-AS1-DHX9 interaction by IL10RB-AS1 RNA pulldown coupled with Western blot. GAPDH acted as negative control. The AML cells were first treated by 5 µM VbP for 24 hours. (**C**) Western blot showing reduced DHX9 protein levels in MV4-11 and MOLM-13 cells 48 hours post-knockdown by siRNA. GAPDH acted as negative control. (**D**) Representative pyroptotic morphology (left) and quantitative analysis(right) for MV4-11 after electrotransfection with si-NC or si-DHX9-1/2 and treatment with 5 µM VbP. Scale bar:20 μm. (**E**) LDH release from AML cells after electrotransfection with si-NC or si-DHX9-1/2 and treatment with 5 µM VbP. (**F**) Flow cytometric analysis of cell death (PI+ / Annexin V+ cells) in AML cells following electrotransfection with si-NC or si-NLRP3-1/2 and subsequent treatment with or without 5 µM VbP. (**G**) RNA pulldown of IL10RB-AS1 with DDX3X from HEK293T cell lysates. Tubulin acted as the negative control. (**H and J**) Immunoblots showing the DDX3X in cells with FLAG-DDX3X overexpression (**H**) or knockdown (**J**). Tubulin or GAPDH acted as the negative control. (**I**) RIP assay using anti-IgG or anti-DDX3X followed by quantitative real-time PCR analysis. GAPDH acted as the negative control. The percentage of RIP-enriched IL10RB-AS1 relative to the input value. (**K**) Representative morphological images of MV4-11 and MOLM-13 cells following DDX3X knockdown. Scale bars: 20 μm. (**L**) Flow cytometric analysis of cell death (PI+ / Annexin V+ cells) in AML cells following electrotransfection with si-NC or si-DDX3X-1/2. (**M, N**) Co-immunoprecipitation (Co-IP) confirms the endogenous interaction between DDX3X and CARD8 from MV4-11 cell lysates. (**O**) Co-IP assay investigates the interaction between DHX9 and CARD8-CT in AML cells. (**P**) RNA pulldown of IL10RB-AS1 with HA-CARD8-CT. Tubulin acted as the negative control. Values are mean ± SD (n = 3), one-way ANOVA in **D, E, F, L**, and unpaired two-tailed Student’s t-test in **I**. ns, not significant; * *P* < 0.05, *** *P* < 0.001.

**Fig. S7. IL10RB-AS1 regulates the VbP-mediated pyroptosis of AML *in vivo***

(**A**) RT-qPCR validation of sh-IL10RB-AS1 knockdown efficiency in MV4-11 cells. Values are mean ± SD (n = 3), and unpaired two-tailed Student’s t-test used. *** *P* < 0.001. (**B**) Representative flow cytometry graphs showing the percentage of hCD45+ indicating MV4-11 proliferation status in the Bone Marrow, eripheral blood, spleen, and liver samples of NOD-SCID mice injected with vehicle, sh-NC, and sh-IL10RB-AS1 cells with vehicle or VbP treatment.

**Table S1. Patient demographics and clinicopathologic features.**

| Category | Median (range) | No. (%) |
| --- | --- | --- |
| AML (n = 40) | | |
| Age at diagnosis (years, Median (range)) | 6 (0.17–15) |  |
| Sex |  |  |
| Male | 26 | 65 |
| Female | 14 | 35 |
| WBC count, × 10^9^/L (Median (range)) | 17.5 (1.9- 529.9) |  |
| FAB |  |  |
| M2 | 15 | 37.5 |
| M3 | 7 | 17.5 |
| M4 | 3 | 7.5 |
| M5 | 11 | 27.5 |
| Missing | 4 | 10 |

**Table S2. Mass-spectrum of proteins from tRSA-IL10RB-AS1 pull down.**

| Mass-spectrum of proteins in MOLM-13 | | | | | |
| --- | --- | --- | --- | --- | --- |
| ProteinName | **-10lgP_value** | **#Unique** | **ProteinName** | **-10lgP_value** | **#Unique** |
| HNRPU | 268.04 | 57 | **ACLY** | 84.69 | 3 |
| NUCL | 240.98 | 29 | **K1C24** | 83.4 | 1 |
| HNRPQ | 227.04 | 22 | **AXA2L** | 81.1 | 4 |
| LA | 221.32 | 27 | **DDX5** | 80.38 | 2 |
| G3P | 217.71 | 19 | **TCPZ** | 78.72 | 4 |
| ACTB | 216.98 | 6 | **RTCA** | 78.44 | 4 |
| SYDM | 213.74 | 29 | **TM10C** | 78.23 | 5 |
| MOES | 210.98 | 27 | **AIMP1** | 78.15 | 4 |
| EF1A1 | 202.22 | 10 | **PGK1** | 78.08 | 4 |
| DUS3L | 200.28 | 28 | **PA2G4** | 76.26 | 4 |
| HNRPD | 198.57 | 13 | **GEMI5** | 75.45 | 3 |
| ACTC | 195.44 | 1 | **KPYM** | 73.91 | 2 |
| ILF3 | 190.82 | 17 | **TCPD** | 73.2 | 4 |
| ALDOA | 190.11 | 15 | **BCKD** | 71.64 | 2 |
| XRCC6 | 187.67 | 20 | **CSDE1** | 69.8 | 3 |
| XRCC5 | 185.39 | 26 | **CPSF6** | 69.74 | 3 |
| HSP7C | 179.85 | 15 | **EF2** | 69.5 | 3 |
| SAMH1 | 173.34 | 17 | **PCCA** | 67.06 | 4 |
| SYRC | 171.38 | 19 | **EF1D** | 64.48 | 3 |
| PABP1 | 169.25 | 11 | **GARS** | 63 | 2 |
| DHX9 | 169.1 | 17 | **HNRPK** | 61.94 | 2 |
| EZRI | 169.03 | 7 | **VASP** | 60.89 | 3 |
| FUBP2 | 166.3 | 15 | **ROA1** | 60.1 | 2 |
| ILF2 | 159.24 | 10 | **IGKC** | 59.36 | 2 |
| ROAA | 153.03 | 8 | **NSUN2** | 57.83 | 3 |
| HNRPR | 150.9 | 2 | **YBOX1** | 57.14 | 2 |
| BIP | 148.39 | 9 | **RLA0** | 56.74 | 1 |
| HNRL2 | 147.74 | 12 | **TCPE** | 56.53 | 2 |
| EF1G | 146.2 | 14 | **PDE12** | 55.42 | 1 |
| PABP2 | 144.87 | 6 | **PUM2** | 54.46 | 1 |
| DDX3X | 142.16 | 3 | **DBR1** | 53.98 | 1 |
| MAOM | 141.02 | 8 | **ROA2** | 51 | 2 |
| DDX3Y | 137.19 | 2 | **PNPT1** | 50.88 | 1 |
| CISY | 136.85 | 7 | **ODO2** | 50.79 | 3 |
| ATPB | 136.69 | 8 | **TRI56** | 49.62 | 2 |
| TRM2A | 136.32 | 14 | **SYDC** | 49.07 | 2 |
| TBB5 | 136.11 | 7 | **IF2B** | 48.87 | 1 |
| HS90B | 135.96 | 4 | **TBA3E** | 48.65 | 2 |
| HNRH1 | 135.69 | 11 | **AIMP2** | 47.91 | 2 |
| IF2A | 134.64 | 9 | **TIF1B** | 46.53 | 3 |
| IDHP | 132.8 | 9 | **DSC1** | 45.4 | 1 |
| TCPQ | 131.98 | 10 | **EWS** | 45.27 | 2 |
| SYYC | 131.15 | 12 | **DDX1** | 45.23 | 2 |
| IF2B1 | 131.11 | 7 | **CH60** | 42.64 | 2 |
| HS90A | 129.55 | 2 | **IGG1** | 42.08 | 2 |
| ARP2 | 128.45 | 8 | **TRI25** | 41.21 | 1 |
| PUS7 | 126.11 | 8 | **MDHM** | 40.75 | 2 |
| TCPH | 125.31 | 12 | **LDHB** | 39.72 | 1 |
| ENOA | 124.85 | 8 | **ROA3** | 39.54 | 1 |
| LRC59 | 121.46 | 5 | **ST134** | 37.23 | 1 |
| ARP3 | 120.18 | 10 | **ERI1** | 37.04 | 2 |
| PABP4 | 117.14 | 2 | **FMNL1** | 36.47 | 1 |
| SYK | 114.99 | 11 | **MMS22** | 34.74 | 1 |
| PLSL | 114.08 | 8 | **H2AZ** | 34.72 | 1 |
| MTREX | 113.01 | 5 | **KPRP** | 34.46 | 2 |
| HNRPF | 112.99 | 4 | **DACT1** | 33.11 | 1 |
| IF2G | 112.52 | 8 | **BASP1** | 32.88 | 1 |
| IF2B2 | 109.41 | 5 | **MYH16** | 32.17 | 1 |
| ALDOC | 107.51 | 1 | **RL40** | 31.64 | 1 |
| ATPA | 105.38 | 6 | **MET16** | 31.52 | 1 |
| K2C78 | 104.01 | 1 | **DESP** | 30.21 | 1 |
| RPN1 | 103.34 | 5 | **AKP13** | 29.32 | 1 |
| FUBP1 | 103.18 | 7 | **TOP2B** | 28.41 | 1 |
| DCD | 99.66 | 4 | **P53** | 26.23 | 1 |
| HNRDL | 97.35 | 1 | **SETX** | 25.98 | 1 |
| TCPA | 93.61 | 7 | **MRUPP** | 23.75 | 1 |
| SYTC | 93.5 | 6 | **NID1** | 22.64 | 1 |
| ODB2 | 87.79 | 6 | **NAR4** | 22.47 | 1 |
| MCCB | 87.36 | 3 | **AOXA** | 22.2 | 1 |
| URP2 | 87.04 | 3 | **COR1A** | 22.15 | 1 |
| F120A | 86.54 | 5 | **DMXL1** | 21.79 | 1 |
| GRP75 | 86.1 | 4 | **DHX36** | 21.59 | 1 |
| TRUA | 86.07 | 4 | **IQGA1** | 21.27 | 1 |
| MCCA | 85.71 | 5 | **BCAS1** | 20.92 | 1 |
| FUS | 84.98 | 5 |  |  |  |
| Mass-spectrum of proteins in MV4-11 | | | | | |
| GeneName | **-10lgP_value** | **#Unique** | **GeneName** | **-10lgP_value** | **#Unique** |
| HNRPU | 282.09 | 51 | **ZC3HF** | 57.47 | 2 |
| HNRPQ | 223.93 | 16 | **YTHD2** | 57.12 | 2 |
| DDX21 | 219.36 | 22 | **LRC47** | 56.26 | 1 |
| DHX9 | 203.28 | 26 | **KINH** | 56.2 | 1 |
| NUCL | 191.94 | 17 | **P4HA1** | 56.19 | 1 |
| PCCA | 189.21 | 18 | **I2BP2** | 56.12 | 1 |
| HNRPM | 189.06 | 24 | **CSTFT** | 55.57 | 2 |
| HNRL2 | 171.59 | 20 | **FUS** | 54.75 | 1 |
| HNRPR | 168.88 | 4 | **AQR** | 54.69 | 2 |
| ALBU | 167.16 | 4 | **RPC2** | 54.54 | 2 |
| ACACA | 165.91 | 22 | **SMRC2** | 54.07 | 2 |
| LPPRC | 158.28 | 16 | **SRRT** | 53.79 | 2 |
| HNRPK | 151.84 | 11 | **TR150** | 53.63 | 1 |
| ZFR | 148.76 | 11 | **HSP7C** | 51.76 | 3 |
| PYC | 148.65 | 11 | **STK10** | 51.39 | 1 |
| PTN6 | 147.13 | 12 | **BTK** | 50.16 | 2 |
| LMNB1 | 145.57 | 8 | **DDX42** | 49.89 | 1 |
| LMNA | 142.12 | 10 | **SART3** | 49.78 | 2 |
| TCPE | 141.54 | 12 | **MCCA** | 49.75 | 1 |
| TCPQ | 141.25 | 9 | **SNUT2** | 48.74 | 1 |
| MOES | 139.83 | 9 | **SHIP1** | 48.28 | 1 |
| PABP1 | 138.89 | 5 | **YBOX1** | 47.93 | 1 |
| CPSF1 | 138.76 | 10 | **YBOX2** | 47.93 | 1 |
| FUBP1 | 137.28 | 8 | **YBOX3** | 47.93 | 1 |
| DDX50 | 135.86 | 5 | **XRCC6** | 47.52 | 2 |
| SF3B1 | 133.73 | 9 | **TRI25** | 46.88 | 1 |
| HNRPL | 129.27 | 7 | **PLXB3** | 46.48 | 1 |
| SF3B3 | 127.31 | 10 | **WDR33** | 46.3 | 1 |
| RPN1 | 125.32 | 9 | **DDX24** | 45.45 | 1 |
| SF3A1 | 124.96 | 7 | **AKP13** | 44.99 | 2 |
| PRKDC | 124.07 | 10 | **TBL3** | 44.93 | 1 |
| DDX17 | 123.38 | 5 | **RENT1** | 44.56 | 1 |
| DCD | 121.39 | 6 | **RRP44** | 43.74 | 1 |
| ILF3 | 120.83 | 5 | **HCFC1** | 42.92 | 1 |
| MATR3 | 115.34 | 6 | **DYH8** | 42.72 | 1 |
| SAMH1 | 112.03 | 5 | **SMC4** | 42.04 | 2 |
| PRP8 | 110.65 | 5 | **NOP58** | 41.54 | 1 |
| ACLY | 110.01 | 6 | **TOP2B** | 41.49 | 1 |
| TCPG | 109.59 | 9 | **NOL6** | 41.28 | 1 |
| PSPC1 | 109.09 | 5 | **EWS** | 40.71 | 1 |
| H12 | 104.74 | 1 | **CA167** | 39.94 | 1 |
| CSDE1 | 104.01 | 3 | **RBP2** | 39.12 | 1 |
| SF3B2 | 103.89 | 8 | **DDX54** | 38.72 | 1 |
| PUR9 | 103.68 | 5 | **SSRP1** | 38.71 | 1 |
| GRP75 | 101.99 | 5 | **RRP5** | 38.2 | 1 |
| H14 | 101.58 | 1 | **SRPRA** | 37.67 | 1 |
| H15 | 101.16 | 4 | **EI2BD** | 37.05 | 1 |
| SYEP | 101.01 | 5 | **ARFG2** | 36.04 | 1 |
| PESC | 100.91 | 4 | **GDS1** | 36.03 | 1 |
| TIF1B | 100.24 | 5 | **DIEXF** | 35.94 | 1 |
| ATD3A | 98.84 | 7 | **VP13D** | 35.68 | 1 |
| RFA1 | 98.35 | 7 | **K1109** | 35.01 | 1 |
| ACADV | 97.37 | 5 | **NSUN2** | 34.73 | 1 |
| NU214 | 96.09 | 6 | **4F2** | 34.44 | 1 |
| ZC3HE | 95.77 | 3 | **ARHGI** | 34.05 | 1 |
| MBB1A | 95.75 | 3 | **ABCF1** | 33.79 | 1 |
| PABP4 | 95.75 | 3 | **LAS1L** | 33.54 | 1 |
| DHX30 | 94.47 | 3 | **CHD4** | 33.01 | 1 |
| SF3A3 | 93.75 | 3 | **PNKP** | 32.94 | 1 |
| U5S1 | 93.45 | 5 | **DYN2** | 32.76 | 1 |
| PR40A | 92.52 | 3 | **DYN3** | 32.76 | 1 |
| MTREX | 92.11 | 4 | **ZC3H4** | 32.66 | 1 |
| PUF60 | 91.89 | 4 | **IF4G1** | 31.88 | 1 |
| SYLC | 90.69 | 3 | **DPOD1** | 31.77 | 1 |
| DDX5 | 88.52 | 2 | **SHRM3** | 31.34 | 1 |
| RU17 | 87.17 | 5 | **SYNC** | 30.77 | 1 |
| HNRL1 | 85.89 | 3 | **ADDB** | 30.65 | 1 |
| EIF3A | 85.47 | 4 | **H2B1A** | 30.64 | 1 |
| IF2B2 | 84.68 | 2 | **H2B1B** | 30.64 | 1 |
| RL1D1 | 83.9 | 3 | **H2B1C** | 30.64 | 1 |
| SP16H | 83.5 | 3 | **H2B1D** | 30.64 | 1 |
| CYFP1 | 81.34 | 3 | **H2B1H** | 30.64 | 1 |
| DSRAD | 80.94 | 4 | **H2B1J** | 30.64 | 1 |
| CHERP | 80.78 | 4 | **H2B1K** | 30.64 | 1 |
| SF01 | 80.63 | 3 | **H2B1L** | 30.64 | 1 |
| MYH9 | 78.22 | 2 | **H2B1M** | 30.64 | 1 |
| CPSF6 | 77.13 | 3 | **H2B1N** | 30.64 | 1 |
| FUBP2 | 76.09 | 2 | **H2B1O** | 30.64 | 1 |
| LC7L3 | 75.53 | 2 | **H2B2E** | 30.64 | 1 |
| SF3A2 | 73.69 | 3 | **H2B2F** | 30.64 | 1 |
| RL40 | 72.71 | 2 | **H2BFS** | 30.64 | 1 |
| RS27A | 72.71 | 2 | **WWC2** | 29.77 | 1 |
| UBB | 72.71 | 2 | **SMCA5** | 29.26 | 1 |
| UBC | 72.71 | 2 | **ECM29** | 28.78 | 1 |
| HP1B3 | 71.01 | 2 | **EIF3L** | 28.45 | 1 |
| FETUA | 69.86 | 4 | **PTHB1** | 28.02 | 1 |
| NOP56 | 69.48 | 3 | **DDX3X** | 27.99 | 1 |
| CCAR2 | 68.53 | 3 | **DDX3Y** | 27.99 | 1 |
| FIP1 | 68.11 | 1 | **DDX4** | 27.99 | 1 |
| EF2 | 66.81 | 1 | **RRP12** | 27.23 | 1 |
| RBM39 | 66.15 | 2 | **CH60** | 26.68 | 1 |
| AIFM1 | 65.78 | 3 | **RBM26** | 26.39 | 1 |
| ABCE1 | 65.61 | 2 | **PICAL** | 26.3 | 1 |
| NOM1 | 65.22 | 1 | **NCLN** | 26.03 | 1 |
| NUP98 | 64.03 | 2 | **SRRM1** | 25.59 | 1 |
| PABP2 | 63.65 | 1 | **SRSF4** | 25.24 | 1 |
| SPR2A | 63.09 | 2 | **CTCF** | 25.13 | 1 |
| SPR2B | 63.09 | 2 | **CTCFL** | 25.13 | 1 |
| SPR2D | 63.09 | 2 | **SMC2** | 24.46 | 1 |
| SPR2E | 63.09 | 2 | **NAR4** | 24.05 | 1 |
| SPR2G | 63.09 | 2 | **GTF2I** | 23.97 | 1 |
| SYF1 | 62.21 | 2 | **MEGF6** | 23.77 | 1 |
| AT131 | 61.96 | 2 | **TOM70** | 23.14 | 1 |
| G3BP1 | 60.74 | 1 | **DDX52** | 21.97 | 1 |
| DDX1 | 60.48 | 2 | **LOXH1** | 21.4 | 1 |
| LMNB2 | 60.31 | 1 | **RBM25** | 21.27 | 1 |
| AKAP8 | 60.15 | 3 | **SORC1** | 20.86 | 1 |
| KSYK | 59.47 | 1 | **BRE1A** | 20.56 | 1 |
| TRY1 | 58.04 | 1 | **CLPX** | 20.34 | 1 |

**Table S3. Oligonucleotide sequences in this study.**

| PCR Primers for vector construction | |
| --- | --- |
| LeGO-IL10RB-AS1--XbaI-F | GCTCTAGACAGATGCACACGAGGAAGAGG |
| LeGO-IL10RB-AS1-XhoI-R | CCGCTCGAGTAACACAACAGTACTTTTTTAATACAAACTTG |
| pCDH-CARD8-FL-XbaI-F | GCTCTAGAATGGAAAAAAAGGAGTGTCCAGAAAAGAGT |
| pCDH-CARD8-FL-EcoR1-R | CGGAATTCCAAATTCTGCTGTCTAAGATAGGACACGA |
| pCDH-CARD8-N-EcoR1-R | CGGAATTCGAAGCTGGGGCTTTCCAGGA |
| pCDH-CARD8-C-XbaI-F | GCTCTAGAATGTCTCTGATGGGCATCCTGCTG |
| pCDH-DDX3X-FL-EcoR1-F | CGGAATTCATGAGTCATGTGGCAGTGGAAAATG |
| pCDH-DDX3X-FL-BamHI-R | CGGGATCCGTTACCCCACCAGTCAACCCCC |
| pCDH-DDX3X-ΔN-EcoRI-F | CGGAATTCATGACTGGGATTAATTTTGAGAAATACGATGAC |
| pCDH-DDX3X-ΔC-BamHI-R | CGGGATCCGTTTTCTAACCAAGACGGCACTTCTTG |
| pCDH-DDX3X-ΔH-F1 | TCTAGAGCTAGCGAATTCATGAGTCATGTGGCAGTGGAAAATG |
| pCDH-DDX3X-ΔH-R1 | CTTGTAGTGGTGTTCATAAGCCATGTTGCCTCCAGAAAAGAGTTCC |
| pCDH-DDX3X-ΔH-F2 | ATGGCTTATGAACACCACTACAAG |
| pCDH-DDX3X-ΔH-R2 | ATCTTTGTAATCGGATCCGTTACCCCACCAGTCAACCCCC |
| Primers used in RNA pulldown and RIP | |
| pEASY-Blunt-T7-tRSA-F | TAATACGACTCACTATAGGGAAAAAAAAAAAAAGCCC |
| pEASY-Blunt-T7-tRSA-R | TTTTTTTTTTTTCTGCAGTGGCGCC |
| pEASY-Blunt-tRSA-IL10RB-AS1-F | GCAGAAAAAAAAAAAACAGATGCACACGAGGAAGAGGA |
| pEASY-Blunt-IL10RB-AS1-R | TAACACAACAGTACTTTTTTAATACAAACTTGGTGTGG |
| Primers used in RT-qPCR | |
| qCEBPA-AS1-F | ACTGCTTCGTTTTCGGTCCA |
| qCEBPA-AS1-R | GAATGGGTCCTGCATTTGGC |
| qLINC00899-F | TTGGTCCTGGGCTTCCATAC |
| qLINC00899-R | GGAAGTTTCCAGGGGGAGTG |
| qIL10RB-AS1-F | AGAGGAAGGGATCCTCGCAA |
| qIL10RB-AS1-R | TCCATTGGAGAGCGTACGTG |
| qHOXA-AS4-F | CAAAGCGCTGCGGAGAAAG |
| qHOXA-AS4-R | CACCAGAACTGGTCGGTGAT |
| qRP11-497G19.1-F | CGACCGGGTTATCTGAGAGC |
| qRP11-497G19.1-R | GCTCTGGCGTGTCAAGGTTA |
| qGAPDH-F | GACAGTCAGCCGCATCTTCT |
| qGAPDH-R | GCGCCCAATACGACCAAATC |
| qGSDMD-F | GTGTGTCAACCTGTCTATCAAGG |
| qGSDMD-R | CATGGCATCGTAGAAGTGGAAG |
| qIL10RB-F | TACCACCTCCCGAAAATGTCA |
| qIL10RB-R | CCCAGTCTGAATGCTCATCTG |
| qIFNAR-F | TCATGGTGTATATCAGCCTCGT |
| qIFNAR-R | AGTTGGTACAATGGAGTGGTTTT |
| qNEAT1-F | GTGGCTGTTGGAGTCGGTAT |
| qNEAT1-R | TAACAAACCACGGTCCATGA |
| qMT-CO1-F | CTTTTCACCGTAGGTGGCCT |
| qMT-CO1-R | AGTGGAAGTGGGCTACAACG |
| Primers used in RACE assay | |
| 5'-R-1 | CATCAGGCGATGCCGTGCATTACCC |
| 5'-R-2 | CGTGCATTACCCAAGACACTGGACA |
| 3'-F-1 | GATCCGGAGCTGTCCAGTGTCTTG |
| 3'-F-2 | CTGTCCAGTGTCTTGGGTAATGCACG |
| shRNA | |
| sh-IL10RB-AS1-1-s | GATCCCCAATGGAAGACAACTCAATTCAAGAGATTGAGTTGTCTTCCATTGGTTTTTG |
| sh-IL10RB-AS1-1-ats | AATTCAAAAACCAATGGAAGACAACTCAATCTCTTGAATTGAGTTGTCTTCCATTGGG |
| sh-IL10RB-AS1-2-s | GATCCCGCTAGAACACCACGTAAATTCAAGAGATTTACGTGGTGTTCTAGCGTTTTTG |
| sh-IL10RB-AS1-2-ats | AATTCAAAAACGCTAGAACACCACGTAAATCTCTTGAATTTACGTGGTGTTCTAGCGG |
| siRNA | |
| si-IL10RB-AS1-1 | CCAAUGGAAGACAACUCAATT |
| si-IL10RB-AS1-2 | CGCUAGAACACCACGUAAATT |
| si-DDX3X-1 | CCUGAACUCUUCAGAUAAUTT |
| si-DDX3X-2 | GCUGGCUCGUGAUUUCUUATT |
| si-NLRP3-1 | GCUGCUGAAAUGGAUUGAATT |
| si-NLRP3-2 | GUGCGUUAGAAACACUUCATT |
| si-CARD8-1 | GGAUGUUGAGUUGAUUGAUTT |
| si-CARD8-2 | GCAGAUGAAGGAACCCAUUTT |
| si-DHX9-1 | GGGCUAUAUCCAUCGAAAUUUTT |
| si-DHX9-2 | ACGACAAUGGAAGCGGAUAUATT |

**Table S4.** **Information of antibodies and chemicals used in this study.**

| REAGENT or RESOURCE | SOURCE | IDENTIFIER |
| --- | --- | --- |
| anti-GSDMD | Abcam | Cat# ab215203; RRID:AB_2916166 |
| anti-PARP | CST | Cat#9542; RRID: AB_2160739 |
| anti-caspase1 | Proteintech | Cat# 22915-1-AP; RRID:AB_2876874 |
| anti CARD8 | Abcam | Cat# ab194585; RRID:AB_3718163 |
| anti-CARD8 | Affinity Biosciences | Cat# DF6092; RRID:AB_2838060 |
| anti-DDX3X | Proteintech | Cat# 67915-1-Ig; RRID:AB_2918669 |
| anti-FLAG | Sigma Aldrich | Cat# F1804; RRID: AB_262044 |
| anti-HA | CST | Cat# 3724; RRID: AB_1549585 |
| anti-HA | Abmart | Cat#M20003; RRID: AB_2864345 |
| anti-GAPDH | Proteintech | Cat# 10494-1-AP; RRID: AB_2263076 |
| anti-ACTIN | TransGen Biotech | Cat#HC201; RRID: AB_2860007 |
| anti-Tubulin | Invitrogen | Cat# 32-2600; RRID: AB_86547 |
| anti-DHX9 | Elabscience | Cat# EPP12284 |
| Normal Mouse IgG | Merck millipore | Cat# 12-371; RRID:AB_145840 |
| Normal Rabbit IgG | Merck millipore | Cat# 12-370; RRID:AB_145841 |
| hCD45 | Invitrogen | Cat# 11-0459-42; RRID: AB_10852703 |
| AlexaFluor 488-conjugated  secondary antibodies(mouse) | Invitrogen | Cat# A28175; RRID: AB_2536161 |
| AlexaFluor 594-conjugated  secondary antibodies (rabbit) | Invitrogen | Cat# A21207; RRID: AB_141637 |
| mouse HRP-conjugated  secondary antibody | CST | Cat# 7076; RRID: AB_330924 |
| rabbit HRP-conjugated secondary antibody | Thermo Fisher Scientific | Cat# 31460; RRID: AB_228341 |
| Talabostat (Val-boroPro) | Selleck | Cat#S8455 |
| Belnacasan（VX-765） | Selleck | Cat# S2228 |
